# Antibody reactive immunomes of *Ehrlichia chaffeensis* and *E. canis* are diverse and defined by conformational antigenic determinants

**DOI:** 10.3389/fcimb.2023.1321291

**Published:** 2024-01-09

**Authors:** Tian Luo, Jignesh G. Patel, Xiaofeng Zhang, Jere W. McBride

**Affiliations:** ^1^ Department of Pathology, University of Texas Medical Branch, Galveston, TX, United States; ^2^ Department of Microbiology and Immunology, University of Texas Medical Branch, Galveston, TX, United States; ^3^ Center for Biodefense and Emerging Infectious Diseases, University of Texas Medical Branch, Galveston, TX, United States; ^4^ Sealy Institute for Vaccine Sciences, University of Texas Medical Branch, Galveston, TX, United States; ^5^ Institute for Human Infections and Immunity, University of Texas Medical Branch, Galveston, TX, United States

**Keywords:** *Ehrlichia*, antibody epitope, conformation-dependent, immunome, immunomics

## Abstract

For decades, the defined antibody reactive proteins of *Ehrlichia chaffeensis* and *E. canis* were limited to a small group with linear antibody epitopes. Recently, our laboratory has utilized an immunomics-based approach to rapidly screen and identify undefined *Ehrlichia chaffeensis* and *E. canis* antigenic proteins and antibody epitopes. In this study, we analyzed the remaining portion (~50%) of the *E. chaffeensis* and *E. canis* proteomes (*n* = 444 and *n =* 405 proteins, respectively), that were not examined in previous studies, to define the complete immunomes of these important pathogens. Almost half of the *E. chaffeensis* proteins screened (196/444) reacted with antibodies in convalescent HME patient sera, while only 43 *E. canis* proteins reacted with CME dog sera. New major immunoreactive proteins were identified in *E. chaffeensis* (*n* = 7) and *E. canis* (*n* = 1), increasing the total number of *E. chaffeensis* (n = 14) and *E. canis* proteins (n = 18) that exhibited antibody reactivity comparable to well-defined major antigenic proteins (TRP120 and TRP19). All of the *E. chaffeensis* but only some *E. canis* major immunoreactive proteins contained major conformation-dependent antibody epitopes. The *E. chaffeensis* immunoreactive proteins were generally small (< 250 amino acids; ~27kDa) and the *E. canis* proteins were slightly larger (> 320 amino acids; ~35 kDa). The majority of these new *Ehrlichia* major immunoreactive proteins were predicted to be type I secreted effectors, some of which contained transmembrane domains. Characterization of the immunomes of *E. chaffeensis* and *E. canis* and understanding the host specific *Ehrlichia* immune responses will facilitate identification of protective antigens and define the biophysical epitope characteristics vital to effective vaccine development for the ehrlichioses.

## Introduction

*Ehrlichia* spp. are tick-transmitted, obligate intracellular bacteria that cause disease in animals and humans, ranging from mild to severe and life-threatening (Paddock and Childs, 2003; McBride and Walker, 2011). *E. chaffeensis* is the etiologic agent of human monocytotropic ehrlichiosis (HME), an emerging zoonosis. *E. canis* is the etiologic agent of canine monocytic ehrlichiosis (CME), a highly prevalent globally distributed hemorrhagic disease in dogs (Paddock and Childs, 2003; Harrus and Waner, 2011).

Conventional immunoblotting approaches have helped identify major antibody reactive proteins from *E. chaffeensis* and *E. canis*, including major outer membrane proteins (OMPs) (Ohashi et al., 1998a; Ohashi et al., 1998b), tandem repeat proteins (TRPs) (Doyle et al., 2006; McBride et al., 2007; Luo et al., 2008; Luo et al., 2009; McBride et al., 2011) and ankyrin repeat proteins (Anks) (Nethery et al., 2007; Luo et al., 2010), that contain linear antibody epitopes. Many of these proteins elicit protective immune responses in *Ehrlichia* infection models (Li et al., 2001; Crocquet-Valdes et al., 2011; Kuriakose et al., 2012; Nambooppha et al., 2022). Moreover, experimental studies have demonstrated protection against infection using live-attenuated vaccines and subunit vaccines (Rudoler et al., 2012; McGill et al., 2016; Budachetri et al., 2022); however, there are no commercial human or veterinary vaccines available for HME or CME.

Until recently, the number of defined *E. chaffeensis* and *E. canis* antigenic proteins has been limited (McBride and Walker, 2010), compared to a large number of antigenic proteins (~7-20% of the proteome) identified in other pathogens, such as *Chlamydia*, *Coxiella, Burkholderia* and *Bartonella* (Barbour et al., 2008; Felgner et al., 2009; Vigil et al., 2010a; Vigil et al., 2010b; Cruz-Fisher et al., 2011; Vigil et al., 2011). Since the completion of whole-genome sequencing of the first bacterium *Haemophilus influenzae* in 1995, a variety of multiomics approaches are now available, including genomics, proteomics, transcriptomics, and immunomics which integrates these omics approaches to study the immunome (Sette et al., 2005; Loman and Pallen, 2015; De Sousa and Doolan, 2016; Babu and Snyder, 2023). The immunome is defined as the set of antigens or epitopes that interface with the host immune system. Genome-based *in silico* reverse vaccinology has also accelerated the identification of new vaccine candidates, but it largely relies on the accuracy of a serial of computational prediction tools, which are limited by the validated protective antigens in which the algorithm is based (Bidmos et al., 2018).

Intracellular bacteria *E. chaffeensis* and *E. canis* have relatively small genomes (1.2 Mbp and 1.3Mbp, respectively) that encode less than 1000 proteins (Dunning Hotopp et al., 2006; Mavromatis et al., 2006). In recent years, access to commercial gene synthesis and cloning has made experimental screening of antigenic proteins feasible. Therefore, we established a high-throughput immunomics-based antigen discovery approach to rapidly identify undiscovered antigenic proteins from *E. chaffeensis* and *E. canis* (Luo et al., 2020; Luo et al., 2021). Since previous investigations suggested that hypothetical proteins were potential antigens, our initial studies prioritized hypothetical proteins and a group of annotated proteins with high antigenicity scores predicted by ANTIGENpro, a sequence-based predictor of protein antigenicity (Magnan et al., 2010). About half of the proteins in the *E. chaffeensis* and *E. canis* proteome have been screened, and many new immunoreactive ehrlichial proteins were identified (Luo et al., 2020; Luo et al., 2021). Most of the recently discovered *Ehrlichia* immunoreactive proteins were predicted to be secreted effector proteins with antibody epitopes that exhibit complete or partial conformation dependence (Luo et al., 2020; Luo et al., 2021).

In this study, we used our established immunomics-based strategy to screen the remaining proteins (~50%) in the *E. chaffeensis* and *E. canis* proteomes. This comprehensive screening provides a detailed analysis of the antibody-reactive immunomes of *E. chaffeensis* and *E. canis* 30 years after *E. chaffeensis* GroEL was identified as the first antibody-reactive protein in 1993 (Sumner et al., 1993). The comprehensive identification and analysis of these *Ehrlichia* spp. immunomes reported herein will accelerate diagnostic, vaccine, and immunotherapeutic development for human and canine ehrlichiosis.

## Materials and methods

### Gene synthesis and cloning

*E. chaffeensis* (Arkansas strain) and *E. canis* (Jake strain) gene sequences are available in the Integrated Microbial Genomes (IMG) (https://img.jgi.doe.gov/) (Chen et al., 2019) and GenBank (https://www.ncbi.nlm.nih.gov/genbank). *Ehrlichia* genes were codon-optimized, chemically synthesized and cloned into pIVEX2.3d vector (containing a 6×His-tag sequence) by Twist Bioscience (San Francisco, CA) or GenScript (Piscataway, NJ). Plasmids were transformed into *Escherichia coli* to amplify, then extracted and lyophilized by the manufacturer.

### *In vitro* transcription and translation (IVTT)

*In vitro* expression of *Ehrlichia* proteins was performed using the NEBExpress cell-free *E. coli* protein synthesis system (New England Biolabs, Ipswich, MA). Lyophilized plasmids were reconstituted in water and purified using the UltraClean 96 PCR cleanup kit (Qiagen, Germantown, MD). Plasmids were then added to *E. coli* extract and a reaction premix in a 96-well plate and incubated at 37°C for 3 h with orbital shaking (300 rpm) according to the manufacturer’s instructions.

### HME and CME antisera

HME patient sera were kind gifts from the Centers for Disease Control and Prevention (Atlanta, GA), Vanderbilt University School of Medicine (Nashville, TN), Washington University and the St. Louis Children’s Hospital (St. Louis, MO). CME sera were obtained from naturally infected dogs from the United States and Colombia. All sera were confirmed to be positive against *E. chaffeensis* or *E. canis* by both indirect fluorescent-antibody assay (IFA) and enzyme-linked immunosorbent assay (ELISA). To avoid reactions with non-specific polyreactive IgM antibodies, which have been previously described in humans (Jones et al., 2012), assays were performed with convalescent sera and bound antibody detected with anti-IgG (H+L) secondary antibodies.

### Dot immunoblot

The expression of *Ehrlichia* proteins by IVTT was confirmed by dot immunoblot with horseradish peroxidase (HRP)-labeled mouse anti-His tag monoclonal antibody (1:500; GenScript) as described previously (Luo et al., 2020). The immunoreactivity of native and denatured proteins was also examined by dot immunoblot using IVTT-expressed proteins purified by MagneHis protein purification system (Promega, Madison, WI) according to the manufacturer. Immunoblots were probed with either HME or CME serum (1:200) and developed with TMB 1-component substrate (Kirkegaard & Perry Laboratories, Gaithersburg, MD).

### ELISA immunoscreening

The immunoreactivity of *Ehrlichia* IVTT-expressed proteins was performed by capturing His-tagged IVTT proteins on an ELISA plate coated with anti-His tag antibody as previously described with minor modifications (Luo et al., 2020). ELISA was performed with HME or CME sera (1:200) and bound antibody detected with alkaline phosphatase-labeled rabbit anti-human IgG (H+L) (1:7,000; Abcam, Cambridge, MA) or anti-dog IgG (H+L) secondary antibodies (1:5000) and BluePhos substrate (Kirkegaard & Perry Laboratories). Dilution buffer containing 4 M urea was used to denature IVTT-expressed proteins and the diluted protein was incubated for 10 min at 99°C before cooling on ice and coating the plate (or membrane for dot blot). Optical density was measured at 650 nm (OD_650_) on a SpectraMax iD5 plate reader (Molecular Devices, Sunnyvale, CA) and OD_650_ values represent the mean reading from 3 wells (± standard deviation) after negative control (IVTT negative protein control and a normal human or canine serum control) background subtraction. A sample ELISA OD_650_ value of ≥ 0.2 was considered positive and ≥ 0.5 a strong positive after subtracting the negative control OD_650_ reading (background). The proteins with mean ELISA OD_650_ of > 1.0 from multiple sera were considered immunodominant and proteins with mean ELISA OD_650_ of 0.5~1.0 subdominant.

### Peptide ELISA

To identify linear antibody epitopes, ELISAs were performed using overlapping peptides (17-23 amino acids; 6 amino acid overlap) (Luo et al., 2009). All peptides were commercially synthesized and supplied as a lyophilized powder (GenScript) and resuspended in molecular biology grade water (1 mg/ml). A small amount of NH_4_OH, acetic acid or dimethyl sulfone was added to help dissolve some acidic, basic, or hydrophobic peptides, respectively, according to peptide solubility guidelines from the manufacturer.

### IFA

The antibody titers in sera from HME patients and CME dogs were determined by IFA as previously described (Luo et al., 2020). Antigen slides were prepared from *E. chaffeensis* (Arkansas)-infected THP-1 cells or *E. canis* (Jake)-infected DH82 cells. Slides were examined with a BX61 epifluorescence microscope (Olympus, Japan).

### Bioinformatic analysis

Online bioinformatic prediction tools used in this study include ANTIGENpro (http://scratch.proteomics.ics.uci.edu), TMHMM 2.0 (https://services.healthtech.dtu.dk/service.php?TMHMM-2.0), SignalP 6.0 (https://services.healthtech.dtu.dk/service.php?SignalP-6.0), SecretomeP 2.0 (https://services.healthtech.dtu.dk/service.php?SecretomeP-2.0), S4TE 2.0 (https://sate.cirad.fr), and PREFFECTOR (http://draco.cs.wpi.edu/preffector).

## Results

### *E. chaffeensis* and *E. canis* immunomics-based screening

Previously, we analyzed the predicted *E. chaffeensis* (Arkansas strain) and *E. canis* (Jake strain) ORFs in both databases of Integrated Microbial Genomes (IMG) and GenBank. After RNA genes, pseudogenes and short ORFs (coded proteins < 42 aa) were excluded, the total number of ORFs in *E. chaffeensis* and *E. canis* genome was determined to be 882 and 928, respectively (Luo et al., 2021). The predicted antigenicity of all proteins was determined using ANTIGENpro and respective antigenicity scores obtained (between 0 and 1). We previously investigated the immunoreactivity of all proteins distributed in the top 350 (with antigenicity scores > ~0.6), excluding previously characterized antigens (such as TRPs and OMPs). In addition, we also previously prioritized hypothetical proteins (including proteins with domain of unknown function [DUF]) regardless of ANTIGENpro rank (Luo et al., 2020; Luo et al., 2021) (**Supplementary Figure 1**).

**Figure 1:**
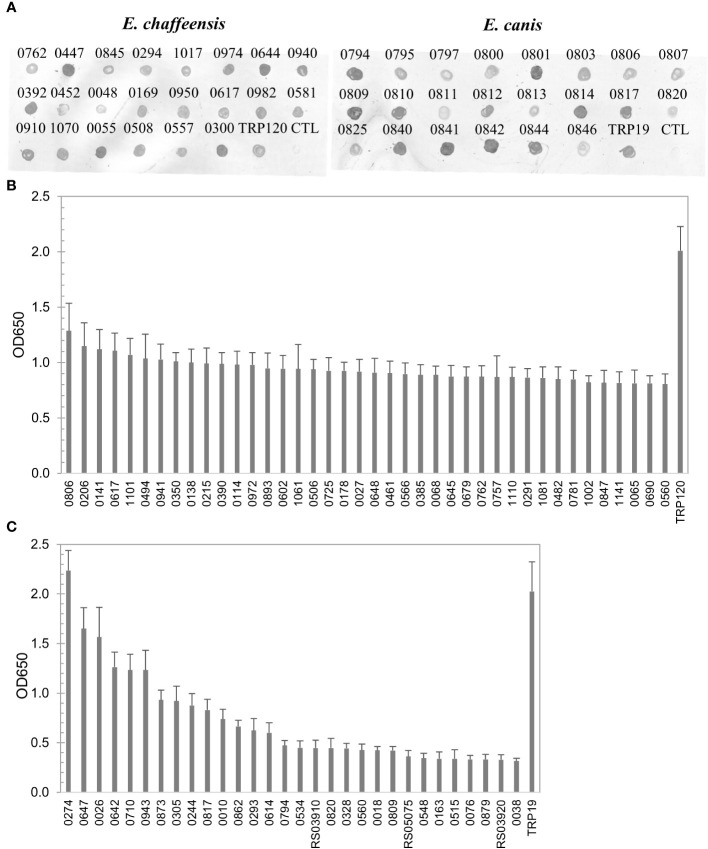
Expression of *E. chaffeensis* and *E. canis* proteins by IVTT and immunoreactivity screening by ELISA. **(A)** Detection of IVTT expression of selected proteins of *E. chaffeensis* and *E. canis* by dot immunoblot with anti-His-tag antibody. CTL, the negative control (IVTT reaction without plasmid template). Pooled sera of HME patients or CME dogs were used to screen *E. chaffeensis* **(B)** and *E. canis* **(C)** proteins, respectively. ELISA OD values represent the mean optical density reading from 3 wells (± standard deviation) after background subtraction. A sample OD of ≥0.2 was considered positive and ≥0.5 a strong positive after subtracting negative control (an IVTT reaction with empty plasmid template and a normal human or canine serum control) readings. TRP120 **(B)** and TRP19 **(C)** were used as positive controls.

To define the complete antigenic repertoire of *E. chaffeensis* and *E. canis*, in this study we further investigated the remaining proteins (*n* = 444 and *n* = 405, respectively) that were not examined in our previous studies. Proteins were expressed in the cell-free IVTT system, and the expression was confirmed by dot blot of randomly selected proteins (*n* = 22) from both *E. chaffeensis* and *E. canis* (**Figure 1A**). Since ELISA plate wells can be saturated by IVTT-expressed proteins, the differences in expression levels did not influence the relative immunoreactivity between different proteins as established in our previous investigations (Luo et al., 2020; Luo et al., 2021).

The *E. chaffeensis* and *E. canis* proteins (*n* = 444 and n=405, respectively) were screened for immunoreactivity by ELISA using pooled convalescent HME or CME sera, respectively, which had indirect fluorescent-antibody assay (IFA) titers of 1600. A total of 196 (44%) *E. chaffeensis* and 43 (11%) *E. canis* proteins reacted with pooled sera (mean optical density at 650 nm [OD_650_] ≥ 0.2 with background subtracted). All *E. chaffeensis* and *E. canis* proteins were ranked according to ELISA OD value (from high to low) and are listed in **Supplementary Tables 1**, **2**, respectively. The *E. chaffeensis* (*n* = 40; mean OD_650_ > 0.8) and *E. canis* proteins (*n* = 31; mean OD_650_ > 0.3) that exhibited the strongest immunoreactivity with pooled sera are shown in **Figures 1B, C**.

### Identification of *E. chaffeensis* and *E. canis* immunodominant proteins

**Figure 2:**
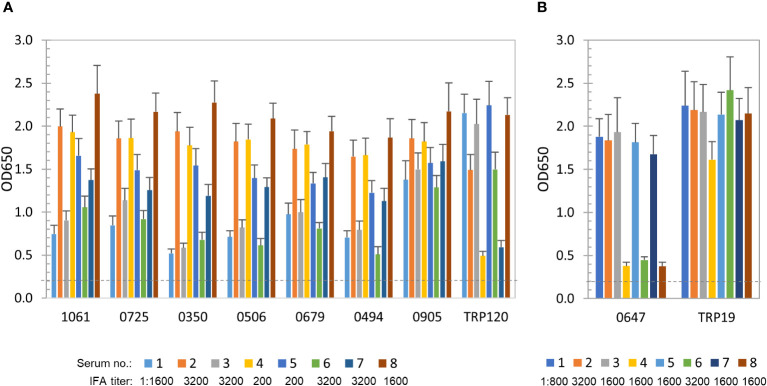
Immunoreactivity of new *E. chaffeensis* and *E. canis* immunodominant proteins. **(A)** Immunoreactivity comparison of 7 *E. chaffeensis* immunodominant proteins and TRP120 by ELISA. IVTT-expressed proteins were probed with a panel of convalescent sera from 8 HME patients. **(B)** Immunoreactivity comparison of *E. canis* immunodominant protein Ecaj_0647 and TRP19 by ELISA. IVTT-expressed proteins were probed with a panel of convalescent sera from 8 CME dogs. OD_650_ values represent the mean optical density reading from 3 wells (± standard deviation) after background subtraction. A sample OD_650_ of ≥ 0.2 was considered positive and ≥ 0.5 a strong positive after subtracting negative control (an IVTT reaction with empty plasmid template and a normal human or canine serum control) readings.

To further define and compare the antibody reactivity of these *E. chaffeensis* and *E. canis* proteins by ELISA, a panel of 8 HME and 8 CME sera were used. All patient and canine sera recognized *E. chaffeensis* or *E. canis* by IFA, respectively, with antibody titers ranging from 200 to 3200 (**Figure 2**). As previously described, well-defined immunodominant proteins (*E. chaffeensis* TRP120 or *E. canis* TRP19) were used as positive controls, respectively (Cardenas et al., 2007; Luo et al., 2010; Pritt and Dumler, 2019; Taques et al., 2020).

*E. chaffeensis* immunoreactive proteins (*n* = 56) were recognized by all or most of 8 HME sera. The top 7 proteins, including Ech_1061, 0725, 0350, 0506, 0679, 0494 and 0905, reacted strongly with all HME sera (similar to TRP120 positive control) and were considered immunodominant (mean OD_650_ values > 1.0) (**Figure 2A**). Additionally, some proteins (*n* = 49) reacted with all sera at lower levels (mean OD_650_ = 0.5~1.0), and only reacted strongly with some HME sera. Thus, these immunoreactive proteins were considered subdominant.

*E. canis* immunoreactive proteins (*n* = 15; mean OD_650_ values of > 0.5) were identified using 8 CME sera, but only 1 (Ecaj_0647) reacted strongly with most canine sera (mean OD_650_ value of > 1.0) and was considered immunodominant (**Figure 2B**). The positive control TRP19 reacted strongly with all CME sera. In addition, 3 *E. canis* proteins (Ecaj_0710, 0026 and 0274) with screening OD_650_ values of > 1.0 exhibited mean OD_650_ values of 0.5-1.0 with 8 CME sera, and thus were considered subdominant.

### Antibody epitopes of immunodominant proteins

**Figure 3:**
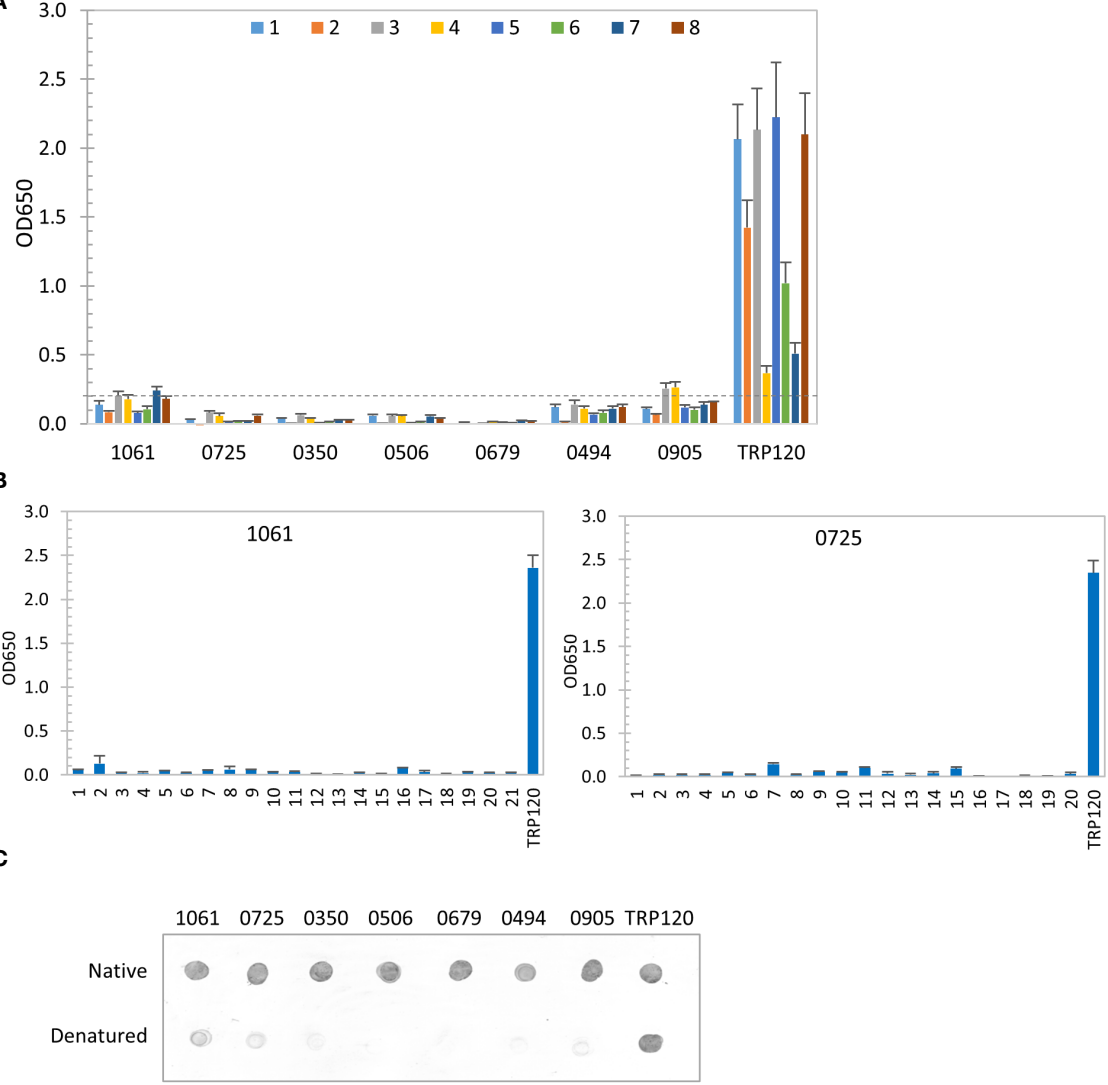
Conformation-dependent immunoreactivity of *E. chaffeensis* immunodominant proteins. **(A)** Immunoreactivity of the denatured IVTT-expressed *E. chaffeensis* proteins compared with TRP120 by ELISA using a panel of 8 HME sera. **(B)** Immunoreactivity of overlapping synthetic peptides spanning 2 *E. chaffeensis* immunoreactive proteins by ELISA with pooled HME sera. Positive control, a TRP120 epitope peptide. OD_650_ values represent the mean optical density reading from 3 wells (± standard deviation). A sample OD_650_ of ≥ 0.2 was considered positive and ≥ 0.5 a strong positive after subtracting negative control (A: an IVTT reaction with empty plasmid template and a normal human or canine serum control; B: a negative peptide) readings. **(C)** Conformation-dependent immunoreactivity of *E. chaffeensis* proteins by dot immunoblot. Immunoreactivity of the native and denatured proteins and TRP120 was detected with serum from an HME patient. All proteins were IVTT expressed and purified.

Recently, we have revealed that most immunoreactive proteins of *E. chaffeensis* and *E. canis* identified previously have conformation-dependent antibody epitopes (Luo et al., 2020; Luo et al., 2021). Thus, in this study we also investigated the conformation-dependence of 7 *E. chaffeensis* and 1 *E. canis* immunodominant proteins by denaturing ELISA. After denaturation using urea, only 2 *E. chaffeensis* immunodominant protein (Ech_1061 and 0905) among top 7 still reacted weakly with 2 HME sera (mean OD_650_ < 0.2 from 8 sera), compared to the native IVTT proteins (mean OD_650_ = 1.25 and 1.01, respectively). The remaining denatured *E. chaffeensis* proteins did not react with any HME patient sera, while the linear epitope-containing major immunoreactive protein control (TRP120) was not affected by denaturation (**Figure 3A**) (McBride and Walker, 2010). These results indicate that these *E. chaffeensis* immunoreactive proteins have conformation-dependent antibody epitopes.

We also used a synthetic peptide ELISA to confirm the absence of major linear epitopes and presence of conformation-dependent epitopes in 2 selected *E. chaffeensis* immunodominant proteins (Ech_1061 and 0725) (Luo et al., 2008; Luo et al., 2009; McBride et al., 2011). Overlapping peptides (17-23 amino acids; 6 amino-acid overlap) covering each entire protein sequence were synthesized. The pooled HME sera used in our initial screening was used to test all peptides by ELISA (**Figure 3B**). None of these peptides reacted with HME sera, demonstrating that these *E. chaffeensis* immunodominant proteins do not contain major linear epitopes, consistent with ELISA results using native and denatured IVTT products (**Figures 2A**, **3A**). We further examined the conformational dependence of epitopes in 7 new *E. chaffeensis* immunodominant proteins by dot immunoblot (**Figure 3C**). The immunoreactivity of native and denatured proteins was compared using an HME serum. After denaturation, these proteins did not react or reacted weakly with *E. chaffeensis* antibodies, consistent with our ELISA data in **Figures 3A, B**. These results support the conclusion that the many immunodominant proteins of *E. chaffeensis* are defined by conformation-dependent antibody epitopes.

**Figure 4:**
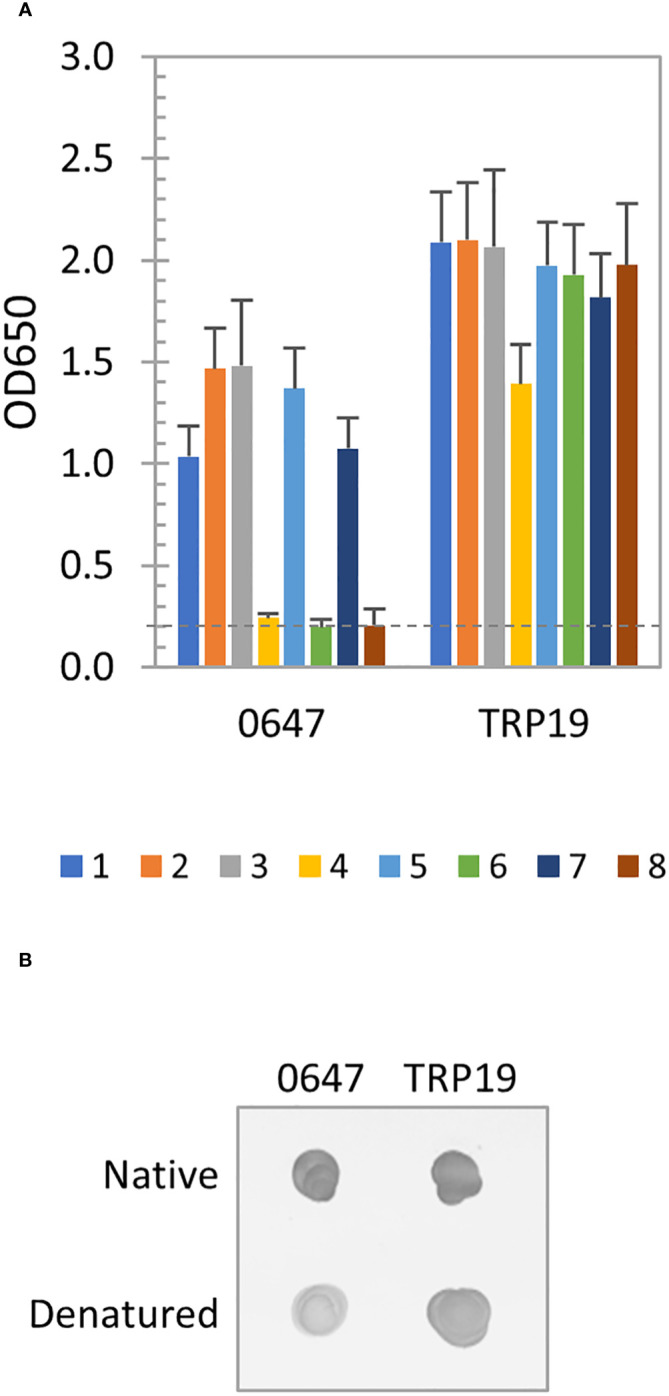
Immunoreactivity of new *E. canis* immunodominant protein. **(A)** Immunoreactivity of the denatured IVTT-expressed Ecaj_0647 protein compared with TRP19 by ELISA using a panel of 8 CME sera. OD_650_ values represent the mean optical density reading from 3 wells (± standard deviation). A sample OD_650_ of ≥ 0.2 was considered positive and ≥ 0.5 a strong positive after subtracting negative control (an IVTT reaction with empty plasmid template and a normal human or canine serum control) readings. **(B)** Immunoreactivity of native and denatured Ecaj_0647 protein compared with TRP19 by dot immunoblot with pooled CME sera. Both proteins were IVTT-expressed and purified.

The immunoreactivity of *E. canis* protein (Ecaj_0647) was only slightly reduced after denaturation by ELISA, indicating a major linear and minor conformation-dependent antibody epitopes were present. The well-defined *E. canis* major immunoreactive protein TRP19 containing a major linear antibody epitope was not affected by denaturation (**Figure 4A**). By dot immunoblot, denatured Ecaj_0647 protein reacted strongly with the *E. canis* antibodies, but at a lower level compared to native proteins, whereas TRP19 protein reacted at a level similar to the native proteins (**Figure 4B**). This result is consistent with our ELISA data in **Figure 4A**.

### Analysis of antigenic proteins

**Figure 5:**
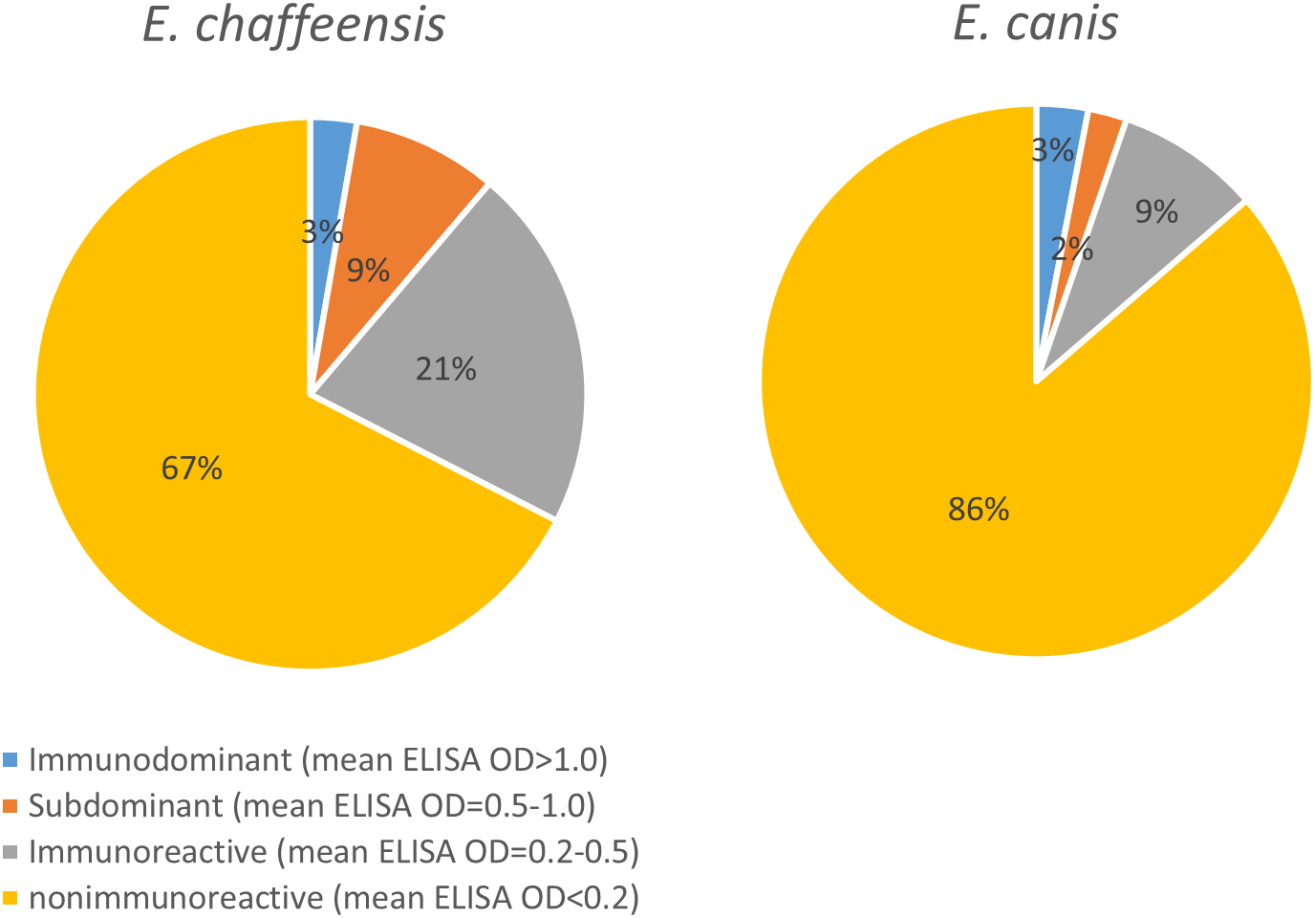
Quantity analysis of antigenic proteins in *E. chaffeensis* and *E. canis* immunomes.

To compile the antigenic repertories of *E. chaffeensis* and *E. canis*, we combined data from this study with our recent studies and summarized the results (**Supplementary Figure 1**). In total, our studies investigated 857 *E. chaffeensis* proteins and 817 *E. canis* proteins, excluding known antigens (such as TRPs and OMPs) and ribosomal proteins, and found 272 (32% of 857) and 112 (14% of 817) immunoreactive proteins, respectively. More importantly, we identified a large number of previously undefined immunodominant proteins in *E. chaffeensis* (*n* = 14) and *E. canis* (*n* = 18). In addition, we identified numerous *Ehrlichia* subdominant proteins (*n* = 70 and *n =* 16, respectively) and other proteins exhibiting low immunoreactivity (*n* = 188 and *n =* 78, respectively) (**Supplementary Figure 1**). **Figure 5** shows the quantity analysis of antigenic proteins in *E. chaffeensis* and *E. canis* immunomes, including previously known antigens. Both *Ehrlichia* immunomes contain ~3% immunodominant proteins; however, *E. chaffeensis* has more subdominant proteins and proteins exhibiting low immunoreactivity than *E. canis* (9% and 21% vs. 2% and 9%, respectively). Therefore, the proportion of the proteome that was not antigenic in *E. canis* and *E. chaffeensis* was 86% and 67%, respectively.

We performed a comprehensive analysis of all new immunodominant proteins of *E. chaffeensis* and *E. canis*. The immunoreactivity, *E. canis* orthologs and bioinformatic analysis of *E. chaffeensis* immunodominant proteins ranked by ELISA OD values are shown in **Tables 1**, **2**. All these proteins contain major conformational epitopes, and a majority of these proteins (*n* = 11) were small (< 250 amino acids) (**Table 1**). Among *E. chaffeensis* immunodominant proteins (*n* = 14), there were 6 hypothetical protein and 8 annotated proteins, including an outer membrane beta-barrel protein, a type IV secretion system component VirB3 and 6 enzymes involved in important biological processes, such as dehydrogenase, transferase, kinase, and phosphatase (**Table 2**). A bioinformatic analysis using multiple online prediction tools found that most *E. chaffeensis* immunodominant proteins (*n* = 8) were predicted to contain at least 1 (up to 8) transmembrane domain by TMHMM 2.0; however, using SignalP 6.0 and SecretomeP 2.0, only 3 proteins (Ech_0745, 0678 and 0679) were predicted to be secreted by a standard secretory signal peptide or a nonclassical (not signal peptide-dependent) protein secretion. Notably, most *E. chaffeensis* immunodominant proteins (*n* = 8) were identified as effectors by PREFFECTOR (Dhroso et al., 2018). Therefore, these proteins were also further analyzed to identify the type of secretion system substrates. Type I and type IV secretion systems (T1SS and T4SS) have been identified in *Ehrlichia*; however, a consensus sequence of type IV secretory motif R-X(7)-R-X-R-X-R (Vergunst et al., 2005) was not identified in any *E. chaffeensis* protein and none of the immunodominant proteins identified in this study were predicted to be type IV substrates by the S4TE 2.0 tool (Noroy et al., 2019). In contrast, a putative type I secretion signal (LDAVTSIF-enriched and KHPMWC-poor) (Delepelaire, 2004; Wakeel et al., 2011) was identified in the last 50 C-terminal residues of these proteins, suggesting that these proteins are type I secreted substrates. Ech_0875 protein showed the greatest difference between the residue occurrences of LDAVTSIF (72%) and KHPMWC (8%) in the last 50 C-terminal amino acids, whereas Ech_0745 showed the least difference (32% versus 18%). These results are consistent with our previous reports and support the conclusion that many of these *E. chaffeensis* immunodominant proteins are type I secreted effectors, although additional experimental validation is required (**Table 2**).

**Table 1:** Immunoreactivity analysis of new *E. chaffeensis* immunodominant proteins.

Protein (Ech_ tag no.)	Mean ELISA OD_650_ *^a^*	Conformational epitope	ANTIGENpro score	*E. canis* ortholog		
				Ecaj_ tag no.	ANTIGENpro score	Immunoreactivity*^b^*
1065	1.91	major	0.70	0857	0.75	++
0578	1.30	major	0.80	*^c^	*	*
1061*^d^*	1.25	major	0.11	0853	0.24	–
0875	1.20	major	0.06	0223	0.17	–
0725*^d^*	1.18	major	0.06	0339	0.10	–
1053	1.15	major	0.76	0846	0.58	–
0207	1.12	major	0.06	0796	0.04	–
0350*^d^*	1.12	major	0.45	0659	0.44	–
0745	1.11	major	0.92	0324	0.92	–
0506*^d^*	1.10	major	0.15	*	*	*
0678	1.07	major	0.54	0369	0.51	–
0679*^d^*	1.03	major	0.13	0368	0.06	–
0494*^d^*	1.03	major	0.10	0534	0.10	+
0905*^d^*	1.01	major	0.27	0200	0.23	–

^a^ Mean OD_650_ from 8 HME patient sera.

^b^ +, immunoreactive in immunoscreening; -, not immunoreactive in immunoscreening; ++, immunodominant.

^c^ *, *E. canis* ortholog not identified.

^d^ Proteins identified in this study. Others were identified in our recent studies.

**Table 2:** Predicted features of new *E. chaffeensis* immunodominant proteins.

Protein (Ech_ tag no.)	Product	No. of AAs/mass (kDa)	Transmembrane domain*^a,b^*	Secretion	T4S*^c^*	Effector*^d^*
1065	2-oxoglutarate dehydrogenase E2 component	404/44	–	–	–	–
0578	Hypothetical protein	185/21	–	–	–	–
1061*^e^*	FMN adenylyltransferase/riboflavin kinase	317/35	–	–	–	+
0875	Phosphatidylglycerophosphatase A	226/25	+	–	–	–
0725*^e^*	Methyltransferase domain-containing protein	264/29	–	–	–	–
1053	Hypothetical protein	193/22	+	–	–	+
0207	Hypothetical protein	176/19	+	–	–	+
0350*^e^*	2-amino-4-hydroxy-6- hydroxymethyldihydropteridine-pyrophosphokinase	169/19	–	–	–	+
0745	Hypothetical protein	118/13	–	+*^f^*	–	+
0506*^e^*	Hypothetical protein	96/11	+	–	–	+
0678	Hypothetical protein	230/25	+	+*^g^*	–	+
0679*^e^*	Outer membrane beta-barrel protein	233/26	+	+	–	+
0494*^e^*	Type IV secretion system protein virb3	97/11	+	–	–	–
0905*^e^*	Phosphatidylglycerophosphatase A	168/18	+	–	–	–

^a^ Predicted by TMHMM.

^b^ +, positive; -, negative.

^c^ Predicted by S4TE.

^d^ Predicted by PREFFECTOR.

^e^ Proteins identified in this study. Others were identified in our recent studies.

^f^ Predicted by SecretomeP.

^g^ Predicted by SignalP.

The analysis of *E. canis* immunodominant protein, ranked ELISA OD values, and comparison with *E. chaffeensis* orthologs are shown in **Tables 3**, **4**. The majority of these proteins (*n* = 14) contained major linear epitopes, including 5 proteins that also contained minor conformational epitopes. Four proteins (Ecaj_0128, 0348, 0857 and 0104) contained only major conformational epitopes (**Table 3**). A majority of *E. canis* immunodominant proteins (*n* = 11) were hypothetical, except for 7 annotated proteins including electron transport protein SCO1/SenC, an extracellular solute-binding protein, translation elongation factor 1A (EF-1A), 2-oxoglutarate dehydrogenase E2 component, peptidyl-prolyl cis-trans isomerase and 2 heat shock proteins (HSP60 and HSP70). Only 8 of these immunodominant proteins were smaller than 250 amino acids. Bioinformatic analysis identified 7 *E. canis* proteins predicted to contain transmembrane domains and 5 proteins that were predicted to be secreted (all by nonclassical mechanism). Importantly, many of *E. canis* immunodominant proteins (*n* = 9) were identified as effectors by PREFFECTOR (Dhroso et al., 2018). Although 3 proteins (Ecaj_0126, 0259 and 0334) were predicted to be type IV substrates by the S4TE 2.0 tool, no type IV secretory signal (R-X[7]-R-X-R-X-R) was identified in any of these *E. canis* proteins. Moreover, a putative type I secretion signal (LDAVTSIF-enriched and KHPMWC-poor) in the C-terminus of these proteins suggested that most of these proteins are type I secreted substrates, consistent with our previous conclusion (Luo et al., 2020; Luo et al., 2021) (**Table 4**). Ecaj_0104 protein had the largest difference between the residue occurrences of LDAVTSIF (70%) and KHPMWC (6%) in the last 50 C-terminal amino acids, whereas the predicted type IV substrate Ech_0259 had the least difference (36% versus 24%).

**Table 3:** Immunoreactivity analysis of new *E. canis* immunodominant proteins.

Protein (Ecaj_ tag no.)	Mean ELISA OD_650_ *^a^*	Conformational epitope	ANTIGENpro score	*E. chaffeensis* ortholog		
				Ech_ tag no.	ANTIGENpro score	Immunoreactivity*^b^*
0919	2.29	no	0.84	1147	0.96	–
0126	2.13	no	0.96	0187	0.97	–
0717	1.85	no	0.80	*^c^	*	*
0151	1.72	no	0.85	0976	0.77	+
0128	1.53	major	0.90	0189	0.88	+
0636	1.52	no	0.76	0377	0.84	–
0073	1.51	no	0.89	0122	0.76	+
0920	1.44	no	0.95	1148	0.85	+
0213	1.44	no	0.61	*	*	*
0259	1.27	minor	0.93	0825	0.92	+
0162	1.25	no	0.60	0960	0.43	–
0348	1.22	major	0.77	*	*	*
0554	1.20	minor	0.84	0471	0.85	–
0857	1.19	major	0.75	1065	0.70	++
0334	1.10	minor	0.87	0731	0.86	+
0647*^d^*	1.08	minor	0.57	0365	0.58	++
0104	1.02	major	0.39	0159	0.77	+
0737	1.00	minor	0.56	*	*	*

^a^ Mean OD_650_ from CME patient sera.

^b^ +, immunoreactive in immunoscreening; -, not immunoreactive in immunoscreening; ++, immunodominant.

^c^ *, *E. chaffeensis* ortholog not identified.

^d^ Proteins identified in this study. Others were identified in our recent studies.

**Table 4:** Predicted features of new *E. canis* immunodominant proteins.

Protein (Ecaj_ tag no.)	Product	No. of amino acids/mass (kDa)	Transmembrane domain*^a,b^*	Secretion*^c^*	T4S*^d^*	Effector*^e^*
0919	Hypothetical protein	120/14	–	+	–	+
0126	Hypothetical protein	671/78	–	+	+	+
0717	Hypothetical protein	226/25	+	–	–	+
0151	Electron transport protein SCO1/senC	205/23	+	–	–	+
0128	Extracellular solute-binding protein, family 1	347/39	+	–	–	+
0636	Hypothetical protein	98/11	–	–	–	–
0073	Hypothetical protein	92/10	–	+	–	–
0920	Hypothetical protein	182/20	–	+	–	+
0213	Hypothetical protein	328/36	+	–	–	–
0259	Hypothetical protein	368/41	–	+	+	+
0162	Translation elongation factor 1A (EF-1A/EF-Tu)	395/43	–	–	–	–
0348	Hypothetical protein	535/59	+	–	–	–
0554	Heat shock protein HSP70	634/69	–	–	–	–
0857	2-oxoglutarate dehydrogenase E2 component	400/44	–	–	–	–
0334	Peptidyl-prolyl cis-trans isomerase	630/72	+	–	+	+
0647*^f^*	chaperonin GroEL (HSP60 family)	552/61	–	–	–	–
0104	Hypothetical protein	182/20	+	–	–	–
0737	Hypothetical protein	194/21	–	–	–	+

^a^ Predicted by TMHMM.

^b^ +, positive; -, negative.

^c^ Predicted by SecretomeP.

^d^ Predicted by S4TE.

^e^ Predicted by PREFFECTOR.

^f^ Proteins identified in this study. Others were identified in our recent studies.

## Discussion

The development of new and affordable biotechniques, such as next-generation genome sequencing, commercial gene synthesis and cloning, and *in vitro* protein expression, has made the analysis of entire bacterial immunomes feasible. Our recent studies have established a rapid high-throughput antigen discovery strategy and we have used this approach to successfully identify many previously undiscovered immunoreactive proteins from *E. chaffeensis* and *E. canis* (Luo et al., 2020; Luo et al., 2021). In this investigation, we identified many new immunodominant and subdominant ehrlichial proteins allowing us to reveal the antibody reactive antigenic repertoires of *E. chaffeensis* and *E. canis.* This information will ultimately expand and accelerate vaccine and diagnostic development for the ehrlichioses.

The application of IVTT in antigen discovery is the key to identification of conformation-dependent immunoreactive proteins, because IVTT generally expresses soluble proteins in native conformation, although posttranslational modifications may not exist (Shimizu et al., 2006; Carlson et al., 2012). The majority of bacterial B-cell epitopes are estimated to be conformational, and many pathogens are known to have immunoreactive proteins with conformational antibody epitopes (Portnyagina et al., 2018; Andrade et al., 2019; Liu et al., 2019; He et al., 2020). However, prior to our studies, the defined *E. chaffeensis* and *E. canis* immunoreactive proteins were limited to those with only major linear epitopes due to the limitations in the approaches used for screening (McBride and Walker, 2010; Lina et al., 2016). Our recent studies have revealed many immunoreactive proteins of *E. chaffeensis* and *E. canis* are predominated by conformation-dependent epitopes (Luo et al., 2020; Luo et al., 2021). Considering the antibody reactive proteins identified in our studies, including the present, all of the new *E. chaffeensis* immunodominant proteins contain major conformation-dependent epitopes; however, linear antibody epitopes are predominant in *E. canis* immunodominant proteins, although we have also identified many conformation-dependent epitopes in immunoreactive protein repertoires of *E. canis*.

Overall, *E. canis* immunodominant proteins appear to have higher ANTIGENpro score and rank than *E. chaffeensis* proteins, which may be related to the differences in the number of linear antibody epitopes found in *E. canis* antigenic proteins (**Tables 1**, **3**). All previously characterized major immunoreactive proteins of *E. chaffeensis* and *E. canis* that contain major linear epitopes, including TRPs, Ank200, OMPs and MSP4, are represented in the top 250 list predicted by ANTIGENpro (Luo et al., 2020), suggesting that the machine learning model of ANTIGENpro may have a bias as it relates to known immunoprotective proteins used to train the algorithm that likely have a predominance of linear epitopes.

Many ehrlichial proteins that were previously considered to have unknown function (hypothetical), including TRPs and Anks, are now known to have defined functions during infection (Wakeel et al., 2009; Luo et al., 2011; Luo and McBride, 2012; Luo et al., 2018; Byerly et al., 2021). Of proteins that make up the *E. chaffeensis* and *E. canis* proteomes, ~25% are considered hypothetical or proteins with domain of unknown function (DUFs). Studies with other intracellular pathogens including our own empirical data in *Ehrlichia* have determined that a large number of these proteins are immunoreactive (Cruz-Fisher et al., 2011; Liu et al., 2019). As a result, we have recently reported that many immunoreactive proteins in the *E. chaffeensis* and *E. canis* proteomes are dominated by hypothetical proteins (Luo et al., 2020; Luo et al., 2021). An analysis of immunodominant proteins in *Ehrlichia* showed that while many hypothetical proteins are *E. chaffeensis* antigenic proteins, more immunoreactive hypothetical proteins exist in *E. canis* (**Tables 2**, **4**).

Among new immunodominant proteins identified in *E. chaffeensis* and *E. canis* in these studies, there were notable proteins with known functions. Interestingly, most *E. chaffeensis* proteins were predicted to be enzymes involved in important biological processes, such as energy production and conversion, coenzyme transport and metabolism, glycerophospholipid metabolism and protein regulation, demonstrating that these metabolically functional *Ehrlichia* proteins are also antigenic. Other more antigenically established proteins included an outer membrane beta-barrel protein and a type IV secretion system protein VirB3 (**Table 2**). Multiple *Ehrlichia*/*Anaplasma* outer membrane proteins and type IV secretion system proteins, such as TRP, OMP, VirB and VirD, have also been previously identified as antigenic and protective (Sutten et al., 2010; Crocquet-Valdes et al., 2011; Kuriakose et al., 2012). Of new immunodominant proteins of *E. canis* identified in these studies, the antigenic annotated proteins are different from those identified in *E. chaffeensis*. For example, a peptidyl-prolyl cis-trans isomerase, an electron transport protein, an extracellular solute-binding protein, a translation elongation factor, and a heat shock protein HSP70 were identified in *E. canis* but not in *E. chaffeensis* (**Table 4**). Another heat shock protein GroEL (HSP60) has an ortholog in *E. chaffeensis* that was identified as the first immunodominant protein in 1993 (Sumner et al., 1993). Immunization with *E. muris* GroEL peptide is protective in a mouse model (Thomas et al., 2011). Notably, many immunodominant proteins of *E. chaffeensis* and *E. canis* were predicted to contain transmembrane domains, further highlighting this feature in many antigenic proteins.

Unlike the previously identified immunoreactive protein orthologs of *Ehrlichia* and *E. canis* that contain major linear epitopes, such as TRPs, Anks and OMPs, only 2 respective *E. canis* or *E. chaffeensis* orthologs (2-oxoglutarate dehydrogenase E2 component and GroEL) in these studies were also found to be immunodominant, and only a minority of *E. chaffeensis* or *E. canis* orthologs shared immunoreactivity, although the respective orthologs were identified for the majority of *Ehrlichia* immunodominant proteins (**Tables 1**, **3**). These findings suggest that *E. chaffeensis* and *E. canis* do not have similar orthologous antigenic proteins as might be expected, and the antibody epitopes in majority of *Ehrlichia* immunodominant proteins are not conserved between *E. chaffeensis* and *E. canis*. Notably, *E. chaffeensis* immunodominant proteins reacted with HME sera more consistently than *E. canis* proteins with CME sera (**Figure 2**). Hence, it is possible that *E. canis* immunoreactive proteins are more antigenically variable among different *E. canis* strains. Antigenic diversity in *E. canis* is well defined, including TRP36, a major immunoreactive protein, and extensive phylogenetic analysis of TRP36 genes has identified several *E. canis* genogroups in North America, Central America, South America, Africa, Europe and Asia, which have antigenic variability (Zhang et al., 2008; Aguiar et al., 2013; Arroyave et al., 2020). Therefore, antigenic variability in some *E. canis* proteins may also contribute to the lower ratio of immunoreactive proteins we found in *E. canis* compared to *E. chaffeensis* in this study and the entire immunomes. In addition, the average size of new *E. chaffeensis* immunodominant proteins appear to be smaller than that of *E. canis*. A vast majority of 14 new *E. chaffeensis* immunodominant proteins (*n* = 11) are small (< 250 amino acids), while only 8 of 18 *E. canis* proteins are small (**Tables 2**, **4**). Collectively, these results indicate the fundamental differences in antigenic protein profiles between *E. chaffeensis* and *E. canis*, which is potentially valuable information for development of specific diagnostics and vaccines for these *Ehrlichia* species.

Consistent with our recent reports (Luo et al., 2020; Luo et al., 2021), the majority of *E. chaffeensis* and *E. canis* immunodominant proteins were predicted to be type I secreted effectors, despite that fact that only 8 proteins were predicted to be secreted by SignalP or SecretomeP. This reinforces the conclusion that in addition to previously defined major immunoreactive proteins that have linear epitopes, such as TRPs and Ank200, there are also other proteins with conformational epitopes that appear to be T1SS substrates. These results further support an important role of the T1SS in *Ehrlichia* infection and potentially immunity (Wakeel et al., 2011). We have shown that several ehrlichial T1S substrates (TRPs) play important roles in pathobiology by regulating important cellular processes to promote ehrlichial survival (Dunphy et al., 2013; Lina et al., 2016; Bui et al., 2023).

Collectively, this investigation and combined with our other recent studies, has successfully defined the antigenic proteins contained in the *E. chaffeensis* and *E. canis* proteomes. We expect that this information will provide a defined set of antigens from which a rational vaccine and diagnostic development strategy can be implemented and tested for HME and CME. Further studies are also needed to determine the T-cell epitopes, secretion mechanism and roles of these proteins in ehrlichial pathobiology and immunity.

## Data availability statement

The original contributions presented in the study are included in the article/**Supplementary Material**. Further inquiries can be directed to the corresponding author.

## Ethics statement

Ethical approval was not required for the studies on humans in accordance with the local legislation and institutional requirements because only commercially available established cell lines were used.

## Author contributions

TL: Data curation, Formal Analysis, Investigation, Methodology, Validation, Writing – original draft, Writing – review & editing. JP: Data curation, Formal Analysis, Investigation, Methodology, Writing – review & editing. XZ: Investigation, Methodology, Writing – review & editing. JM: Conceptualization, Data curation, Funding acquisition, Supervision, Writing – review & editing.
