# Supplementary material for: Antibody reactive immunomes of Ehrlichia chaffeensis and E. canis are diverse and defined by conformational antigenic determinants
Source: Front Cell Infect Microbiol. 2024 Jan 9;13:1321291. doi: 10.3389/fcimb.2023.1321291 (PMC10803646; doi:10.3389/fcimb.2023.1321291)
Supplement: Supplementary file 1 [file Presentation_1.pptx]

## Slide 1
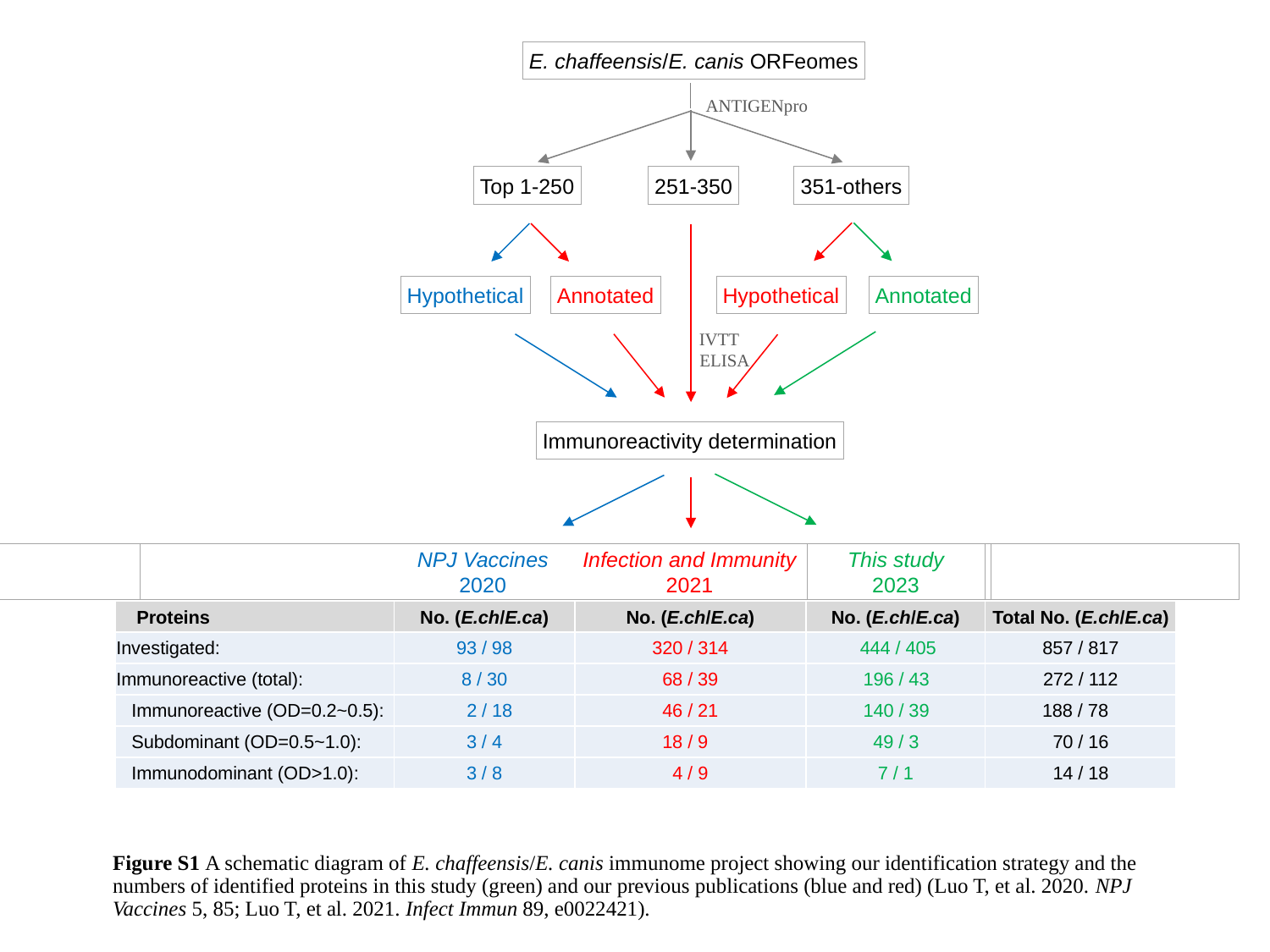

E. chaffeensis/E. canis ORFeomes
ANTIGENpro
Top 1-250
251-350
351-others
Hypothetical
Annotated
Hypothetical
Annotated
IVTT
ELISA
Immunoreactivity determination
NPJ Vaccines
2020
Infection and Immunity
2021
This study
2023
| Proteins | No. (E.ch/E.ca) | No. (E.ch/E.ca) | No. (E.ch/E.ca) | Total No. (E.ch/E.ca) |
| --- | --- | --- | --- | --- |
| Investigated: | 93 / 98 | 320 / 314 | 444 / 405 | 857 / 817 |
| Immunoreactive (total): | 8 / 30 | 68 / 39 | 196 / 43 | 272 / 112 |
| Immunoreactive (OD=0.2~0.5): | 2 / 18 | 46 / 21 | 140 / 39 | 188 / 78 |
| Subdominant (OD=0.5~1.0): | 3 / 4 | 18 / 9 | 49 / 3 | 70 / 16 |
| Immunodominant (OD>1.0): | 3 / 8 | 4 / 9 | 7 / 1 | 14 / 18 |
Figure S1 A schematic diagram of E. chaffeensis/E. canis immunome project showing our identification strategy and the numbers of identified proteins in this study (green) and our previous publications (blue and red) (Luo T, et al. 2020. NPJ Vaccines 5, 85; Luo T, et al. 2021. Infect Immun 89, e0022421).
