# Supplementary material for: Antibody reactive immunomes of Ehrlichia chaffeensis and E. canis are diverse and defined by conformational antigenic determinants
Source: Front Cell Infect Microbiol. 2024 Jan 9;13:1321291. doi: 10.3389/fcimb.2023.1321291 (PMC10803646; doi:10.3389/fcimb.2023.1321291)
Supplement: Supplementary file 2 [file Table_1.docx]

**TABLE S1** *E. chaffeensis* protein immunoreactivity ranked by mean ELISA OD values.

| No. | Ech_ tag no. | Mean ELISA OD_650_ | Antigenicity score |
| --- | --- | --- | --- |
| 1 | 0806 | 1.29 | 0.43 |
| 2 | 0206 | 1.15 | 0.23 |
| 3 | 0141 | 1.12 | 0.46 |
| 4 | 0617 | 1.11 | 0.04 |
| 5 | 1101 | 1.07 | 0.04 |
| 6 | 0494 | 1.04 | 0.10 |
| 7 | 0941 | 1.03 | 0.20 |
| 8 | 0350 | 1.01 | 0.45 |
| 9 | 0138 | 1.00 | 0.42 |
| 10 | 0215 | 0.99 | 0.04 |
| 11 | 0390 | 0.99 | 0.34 |
| 12 | 0114 | 0.98 | 0.54 |
| 13 | 0972 | 0.98 | 0.05 |
| 14 | 0893 | 0.95 | 0.36 |
| 15 | 0602 | 0.94 | 0.54 |
| 16 | 1061 | 0.94 | 0.11 |
| 17 | 0506 | 0.94 | 0.15 |
| 18 | 0725 | 0.93 | 0.06 |
| 19 | 0178 | 0.92 | 0.16 |
| 20 | 0027 | 0.92 | 0.08 |
| 21 | 0648 | 0.91 | 0.56 |
| 22 | 0461 | 0.90 | 0.57 |
| 23 | 0566 | 0.90 | 0.19 |
| 24 | 0385 | 0.89 | 0.23 |
| 25 | 0068 | 0.89 | 0.36 |
| 26 | 0645 | 0.87 | 0.60 |
| 27 | 0679 | 0.87 | 0.13 |
| 28 | 0762 | 0.87 | 0.20 |
| 29 | 0757 | 0.87 | 0.26 |
| 30 | 1110 | 0.87 | 0.43 |
| 31 | 0291 | 0.86 | 0.48 |
| 32 | 1081 | 0.86 | 0.44 |
| 33 | 0482 | 0.85 | 0.33 |
| 34 | 0781 | 0.85 | 0.08 |
| 35 | 1002 | 0.82 | 0.15 |
| 36 | 0847 | 0.82 | 0.28 |
| 37 | 1141 | 0.82 | 0.10 |
| 38 | 0065 | 0.81 | 0.11 |
| 39 | 0690 | 0.81 | 0.29 |
| No. | Ech_ tag no. | Mean ELISA OD_650_ | Antigenicity score |
| 40 | 0560 | 0.81 | 0.09 |
| 41 | 0556 | 0.79 | 0.04 |
| 42 | 1067 | 0.78 | 0.55 |
| 43 | 0230 | 0.77 | 0.05 |
| 44 | 0646 | 0.76 | 0.39 |
| 45 | 0523 | 0.76 | 0.59 |
| 46 | 0776 | 0.76 | 0.39 |
| 47 | 0467 | 0.76 | 0.09 |
| 48 | 0842 | 0.75 | 0.57 |
| 49 | 1003 | 0.75 | 0.15 |
| 50 | 0608 | 0.75 | 0.06 |
| 51 | 0441 | 0.74 | 0.16 |
| 52 | 0567 | 0.74 | 0.52 |
| 53 | 1023 | 0.73 | 0.36 |
| 54 | 0072 | 0.73 | 0.17 |
| 55 | 1086 | 0.72 | 0.09 |
| 56 | 0009 | 0.72 | 0.04 |
| 57 | 0331 | 0.72 | 0.26 |
| 58 | 0084 | 0.72 | 0.05 |
| 59 | 0318 | 0.72 | 0.05 |
| 60 | 1063 | 0.72 | 0.28 |
| 61 | 0224 | 0.70 | 0.54 |
| 62 | 0857 | 0.70 | 0.48 |
| 63 | 0179 | 0.70 | 0.06 |
| 64 | 0193 | 0.70 | 0.07 |
| 65 | 1004 | 0.70 | 0.09 |
| 66 | 0428 | 0.69 | 0.05 |
| 67 | 0371 | 0.68 | 0.57 |
| 68 | 0944 | 0.68 | 0.16 |
| 69 | 0964 | 0.68 | 0.58 |
| 70 | 1155 | 0.68 | 0.27 |
| 71 | 1043 | 0.68 | 0.33 |
| 72 | 0373 | 0.68 | 0.59 |
| 73 | 0818 | 0.67 | 0.04 |
| 74 | 0920 | 0.67 | 0.13 |
| 75 | 0769 | 0.65 | 0.10 |
| 76 | 0517 | 0.65 | 0.07 |
| 77 | 0295 | 0.65 | 0.57 |
| 78 | 0973 | 0.65 | 0.32 |
| No. | Ech_ tag no. | Mean ELISA OD_650_ | Antigenicity score |
| 79 | 0095 | 0.65 | 0.08 |
| 80 | 0621 | 0.65 | 0.38 |
| 81 | 0227 | 0.64 | 0.42 |
| 82 | 0148 | 0.64 | 0.15 |
| 83 | 0780 | 0.64 | 0.05 |
| 84 | 0598 | 0.63 | 0.20 |
| 85 | 1060 | 0.63 | 0.06 |
| 86 | 0234 | 0.63 | 0.39 |
| 87 | 0588 | 0.62 | 0.59 |
| 88 | 0294 | 0.62 | 0.22 |
| 89 | 0264 | 0.62 | 0.48 |
| 90 | 0660 | 0.61 | 0.45 |
| 91 | 0779 | 0.60 | 0.25 |
| 92 | 0620 | 0.60 | 0.60 |
| 93 | 1068 | 0.59 | 0.05 |
| 94 | 0212 | 0.59 | 0.06 |
| 95 | 0905 | 0.57 | 0.27 |
| 96 | 0521 | 0.57 | 0.10 |
| 97 | 0029 | 0.57 | 0.13 |
| 98 | 0337 | 0.57 | 0.03 |
| 99 | 0575 | 0.56 | 0.18 |
| 100 | 0401 | 0.56 | 0.53 |
| 101 | 0897 | 0.56 | 0.50 |
| 102 | 0786 | 0.55 | 0.03 |
| 103 | 1017 | 0.55 | 0.12 |
| 104 | 0269 | 0.54 | 0.05 |
| 105 | 0438 | 0.54 | 0.07 |
| 106 | 0902 | 0.53 | 0.55 |
| 107 | 0890 | 0.53 | 0.22 |
| 108 | 0675 | 0.52 | 0.48 |
| 109 | 0435 | 0.52 | 0.13 |
| 110 | 0128 | 0.51 | 0.45 |
| 111 | 0125 | 0.51 | 0.25 |
| 112 | 0328 | 0.51 | 0.10 |
| 113 | 0393 | 0.50 | 0.06 |
| 114 | 1047 | 0.50 | 0.05 |
| 115 | 0636 | 0.50 | 0.12 |
| 116 | 0321 | 0.50 | 0.11 |
| 117 | 0336 | 0.50 | 0.25 |
| 118 | 0035 | 0.49 | 0.21 |
| 119 | 0067 | 0.49 | 0.09 |
| No. | Ech_ tag no. | Mean ELISA OD_650_ | Antigenicity score |
| 120 | 0940 | 0.48 | 0.35 |
| 121 | 0732 | 0.48 | 0.55 |
| 122 | 0709/0713 | 0.47 | 0.16 |
| 123 | 0383 | 0.47 | 0.26 |
| 124 | 0174 | 0.47 | 0.42 |
| 125 | 0063 | 0.46 | 0.15 |
| 126 | 1044 | 0.46 | 0.06 |
| 127 | 0977 | 0.46 | 0.53 |
| 128 | 0552 | 0.45 | 0.06 |
| 129 | 0557 | 0.45 | 0.27 |
| 130 | 0073 | 0.45 | 0.54 |
| 131 | 0307 | 0.45 | 0.11 |
| 132 | 0844 | 0.45 | 0.13 |
| 133 | 0644 | 0.45 | 0.18 |
| 134 | 0643 | 0.44 | 0.55 |
| 135 | 0127 | 0.44 | 0.23 |
| 136 | 1077 | 0.44 | 0.10 |
| 137 | 0323 | 0.44 | 0.15 |
| 138 | 0172 | 0.44 | 0.54 |
| 139 | 0841 | 0.44 | 0.18 |
| 140 | 0338 | 0.43 | 0.31 |
| 141 | 0801 | 0.43 | 0.22 |
| 142 | 0191 | 0.43 | 0.05 |
| 143 | 0782 | 0.43 | 0.35 |
| 144 | 0522 | 0.42 | 0.30 |
| 145 | 0553 | 0.42 | 0.14 |
| 146 | 1087 | 0.41 | 0.06 |
| 147 | 0581 | 0.41 | 0.08 |
| 148 | 0765 | 0.40 | 0.41 |
| 149 | 0382 | 0.39 | 0.10 |
| 150 | 0483 | 0.39 | 0.33 |
| 151 | 0183 | 0.39 | 0.32 |
| 152 | 0892 | 0.39 | 0.42 |
| 153 | 0548 | 0.39 | 0.28 |
| 154 | 0741 | 0.39 | 0.52 |
| 155 | 0728 | 0.38 | 0.49 |
| 156 | 1012 | 0.38 | 0.43 |
| 157 | 0840 | 0.37 | 0.34 |
| 158 | 0492 | 0.37 | 0.18 |
| 159 | 0554 | 0.37 | 0.11 |
| 160 | 1079 | 0.37 | 0.16 |
| No. | Ech_ tag no. | Mean ELISA OD_650_ | Antigenicity score |
| 161 | 0452 | 0.36 | 0.21 |
| 162 | 1084 | 0.36 | 0.27 |
| 163 | 0885 | 0.35 | 0.23 |
| 164 | 0740 | 0.34 | 0.34 |
| 165 | 0737 | 0.34 | 0.23 |
| 166 | 0590 | 0.34 | 0.09 |
| 167 | 0845 | 0.34 | 0.37 |
| 168 | 0508 | 0.33 | 0.39 |
| 169 | 1082 | 0.33 | 0.14 |
| 170 | 0146 | 0.32 | 0.08 |
| 171 | 0918 | 0.32 | 0.07 |
| 172 | 0966 | 0.32 | 0.36 |
| 173 | 1070 | 0.31 | 0.20 |
| 174 | 0474 | 0.31 | 0.06 |
| 175 | 0093 | 0.31 | 0.36 |
| 176 | 0685 | 0.29 | 0.07 |
| 177 | 1098 | 0.28 | 0.46 |
| 178 | 0982 | 0.28 | 0.19 |
| 179 | 0910 | 0.28 | 0.07 |
| 180 | 0085 | 0.28 | 0.19 |
| 181 | 0504 | 0.28 | 0.43 |
| 182 | 0289 | 0.27 | 0.45 |
| 183 | 0299 | 0.26 | 0.26 |
| 184 | 0816 | 0.26 | 0.05 |
| 185 | 0313 | 0.26 | 0.15 |
| 186 | 0496 | 0.25 | 0.17 |
| 187 | 0036 | 0.25 | 0.27 |
| 188 | 0341 | 0.25 | 0.25 |
| 189 | 0882 | 0.24 | 0.40 |
| 190 | 0573 | 0.23 | 0.41 |
| 191 | 0838 | 0.22 | 0.06 |
| 192 | 0055 | 0.22 | 0.41 |
| 193 | 0724 | 0.21 | 0.40 |
| 194 | 0048 | 0.21 | 0.43 |
| 195 | 0974 | 0.21 | 0.23 |
| 196 | 0469 | 0.21 | 0.16 |
| 197 | 0161 | 0.19 | 0.59 |
| 198 | 0909 | 0.19 | 0.04 |
| 199 | 0536 | 0.19 | 0.14 |
| 200 | 1040 | 0.19 | 0.04 |
| 201 | 1097 | 0.19 | 0.51 |
| No. | Ech_ tag no. | Mean ELISA OD_650_ | Antigenicity score |
| 202 | 1028 | 0.19 | 0.05 |
| 203 | 0333 | 0.18 | 0.23 |
| 204 | 0447 | 0.18 | 0.15 |
| 205 | 1115 | 0.18 | 0.43 |
| 206 | 0616 | 0.17 | 0.11 |
| 207 | 0666 | 0.17 | 0.44 |
| 208 | 1088 | 0.17 | 0.51 |
| 209 | 0516 | 0.16 | 0.19 |
| 210 | 0562 | 0.16 | 0.29 |
| 211 | 0510 | 0.16 | 0.55 |
| 212 | 0599 | 0.16 | 0.37 |
| 213 | 0266 | 0.16 | 0.16 |
| 214 | 0392 | 0.15 | 0.29 |
| 215 | 0288 | 0.15 | 0.06 |
| 216 | 0530 | 0.15 | 0.04 |
| 217 | 0169 | 0.15 | 0.22 |
| 218 | 0123 | 0.14 | 0.45 |
| 219 | 0950 | 0.14 | 0.16 |
| 220 | 0334 | 0.14 | 0.45 |
| 221 | 0311 | 0.14 | 0.23 |
| 222 | 1078 | 0.13 | 0.21 |
| 223 | 0537 | 0.13 | 0.32 |
| 224 | 0031 | 0.13 | 0.36 |
| 225 | 1093 | 0.13 | 0.04 |
| 226 | 0305 | 0.13 | 0.36 |
| 227 | 0056 | 0.13 | 0.23 |
| 228 | 0173 | 0.13 | 0.39 |
| 229 | 0571 | 0.13 | 0.21 |
| 230 | 1106 | 0.13 | 0.14 |
| 231 | 0369 | 0.13 | 0.42 |
| 232 | 0702 | 0.12 | 0.14 |
| 233 | 0175 | 0.12 | 0.44 |
| 234 | 0091 | 0.12 | 0.14 |
| 235 | 0751 | 0.12 | 0.22 |
| 236 | 0300 | 0.12 | 0.13 |
| 237 | 0637 | 0.12 | 0.36 |
| 238 | 0852 | 0.12 | 0.33 |
| 239 | 0761 | 0.12 | 0.39 |
| 240 | 1122 | 0.12 | 0.25 |
| 241 | 0439 | 0.12 | 0.37 |
| 242 | 0638 | 0.11 | 0.28 |
| No. | Ech_ tag no. | Mean ELISA OD_650_ | Antigenicity score |
| 243 | 0217 | 0.11 | 0.34 |
| 244 | 0655 | 0.11 | 0.40 |
| 245 | 0459 | 0.11 | 0.45 |
| 246 | 0062 | 0.11 | 0.31 |
| 247 | 0466 | 0.11 | 0.15 |
| 248 | 0629 | 0.11 | 0.26 |
| 249 | 0605 | 0.11 | 0.42 |
| 250 | 0465 | 0.11 | 0.41 |
| 251 | 0211 | 0.11 | 0.12 |
| 252 | 0315 | 0.11 | 0.44 |
| 253 | 0900 | 0.10 | 0.31 |
| 254 | 0701 | 0.10 | 0.47 |
| 255 | 0132 | 0.10 | 0.17 |
| 256 | 1005 | 0.10 | 0.26 |
| 257 | 1006 | 0.10 | 0.37 |
| 258 | 0200/0994 | 0.10 | 0.19 |
| 259 | 0998 | 0.10 | 0.41 |
| 260 | 0543 | 0.10 | 0.57 |
| 261 | 0693 | 0.10 | 0.29 |
| 262 | 0814 | 0.10 | 0.24 |
| 263 | 0561 | 0.10 | 0.36 |
| 264 | 0774 | 0.09 | 0.13 |
| 265 | 0306 | 0.09 | 0.05 |
| 266 | 0688 | 0.09 | 0.35 |
| 267 | 0137 | 0.09 | 0.13 |
| 268 | 0772 | 0.09 | 0.07 |
| 269 | 0339 | 0.09 | 0.18 |
| 270 | 0960 | 0.09 | 0.42 |
| 271 | 0018 | 0.09 | 0.49 |
| 272 | 0346 | 0.09 | 0.36 |
| 273 | 0789 | 0.08 | 0.44 |
| 274 | 0618 | 0.08 | 0.29 |
| 275 | 0032 | 0.08 | 0.49 |
| 276 | 0024 | 0.08 | 0.37 |
| 277 | 0967 | 0.07 | 0.59 |
| 278 | 0344 | 0.07 | 0.17 |
| 279 | 0237 | 0.07 | 0.44 |
| 280 | 0124 | 0.06 | 0.37 |
| 281 | 0811 | 0.06 | 0.44 |
| 282 | 0013 | 0.05 | 0.42 |
| 283 | 1000 | 0.05 | 0.28 |
| No. | Ech_ tag no. | Mean ELISA OD_650_ | Antigenicity score |
| 284 | 0347 | 0.05 | 0.25 |
| 285 | 0746 | 0.05 | 0.40 |
| 286 | 1090 | 0.04 | 0.37 |
| 287 | 0555 | 0.04 | 0.07 |
| 288 | 0135 | 0.04 | 0.47 |
| 289 | 0958 | 0.04 | 0.05 |
| 290 | 0361 | 0.04 | 0.05 |
| 291 | 0362 | 0.04 | 0.43 |
| 292 | 0532 | 0.04 | 0.46 |
| 293 | 0788 | 0.04 | 0.47 |
| 294 | 0858 | 0.04 | 0.38 |
| 295 | 0667 | 0.03 | 0.41 |
| 296 | 0824 | 0.03 | 0.33 |
| 297 | 0367 | 0.03 | 0.29 |
| 298 | 0495 | 0.03 | 0.37 |
| 299 | 0820 | 0.03 | 0.16 |
| 300 | 0952 | 0.03 | 0.21 |
| 301 | 0832 | 0.03 | 0.36 |
| 302 | 0804 | 0.02 | 0.37 |
| 303 | 0951 | 0.02 | 0.39 |
| 304 | 0771 | 0.02 | 0.16 |
| 305 | 0434 | 0.01 | 0.37 |
| 306 | 0400 | 0.01 | 0.07 |
| 307 | 0087 | 0.01 | 0.59 |
| 308 | 0899 | 0 | 0.18 |
| 309 | 0794 | 0 | 0.60 |
| 310 | 0210 | 0 | 0.59 |
| 311 | 0376 | 0 | 0.59 |
| 312 | 0538 | 0 | 0.59 |
| 313 | 0961 | 0 | 0.58 |
| 314 | 0061 | 0 | 0.58 |
| 315 | 0703 | 0 | 0.58 |
| 316 | 0996 | 0 | 0.58 |
| 317 | 0381 | 0 | 0.57 |
| 318 | 0387 | 0 | 0.57 |
| 319 | 0330 | 0 | 0.57 |
| 320 | 0479 | 0 | 0.57 |
| 321 | 0160 | 0 | 0.57 |
| 322 | 0783 | 0 | 0.57 |
| 323 | 0133 | 0 | 0.57 |
| 324 | 0208 | 0 | 0.57 |
| No. | Ech_ tag no. | Mean ELISA OD_650_ | Antigenicity score |
| 325 | 0809 | 0 | 0.56 |
| 326 | 0497 | 0 | 0.55 |
| 327 | 0594 | 0 | 0.55 |
| 328 | 0134 | 0 | 0.55 |
| 329 | 0088 | 0 | 0.55 |
| 330 | 0773 | 0 | 0.54 |
| 331 | 0791 | 0 | 0.54 |
| 332 | 0913 | 0 | 0.54 |
| 333 | 0860 | 0 | 0.54 |
| 334 | 0540 | 0 | 0.54 |
| 335 | 0298 | 0 | 0.53 |
| 336 | 1156 | 0 | 0.53 |
| 337 | 0225 | 0 | 0.53 |
| 338 | 0045 | 0 | 0.53 |
| 339 | 0726 | 0 | 0.53 |
| 340 | 1116 | 0 | 0.52 |
| 341 | 0322 | 0 | 0.52 |
| 342 | 0877 | 0 | 0.52 |
| 343 | 1117 | 0 | 0.52 |
| 344 | 0026 | 0 | 0.52 |
| 345 | 0544 | 0 | 0.52 |
| 346 | 0957 | 0 | 0.52 |
| 347 | 0680 | 0 | 0.51 |
| 348 | 0119 | 0 | 0.51 |
| 349 | 0006 | 0 | 0.50 |
| 350 | 0060 | 0 | 0.50 |
| 351 | 0092 | 0 | 0.50 |
| 352 | 0342 | 0 | 0.50 |
| 353 | 0509 | 0 | 0.50 |
| 354 | 0873 | 0 | 0.50 |
| 355 | 1149 | 0 | 0.50 |
| 356 | 1051 | 0 | 0.49 |
| 357 | 0195 | 0 | 0.49 |
| 358 | 0793 | 0 | 0.49 |
| 359 | 0464 | 0 | 0.49 |
| 360 | 0404 | 0 | 0.49 |
| 361 | 0202 | 0 | 0.48 |
| 362 | 0515 | 0 | 0.48 |
| 363 | 0030 | 0 | 0.48 |
| 364 | 0626 | 0 | 0.48 |
| 365 | 0139 | 0 | 0.48 |
| No. | Ech_ tag no. | Mean ELISA OD_650_ | Antigenicity score |
| 366 | 0058 | 0 | 0.47 |
| 367 | 0020 | 0 | 0.47 |
| 368 | 0001 | 0 | 0.47 |
| 369 | 0651 | 0 | 0.47 |
| 370 | 0455 | 0 | 0.47 |
| 371 | 0502 | 0 | 0.46 |
| 372 | 0528 | 0 | 0.46 |
| 373 | 0542 | 0 | 0.44 |
| 374 | 0449 | 0 | 0.43 |
| 375 | 0805 | 0 | 0.43 |
| 376 | 0082 | 0 | 0.43 |
| 377 | 0003 | 0 | 0.43 |
| 378 | 0766 | 0 | 0.41 |
| 379 | 0473 | 0 | 0.40 |
| 380 | 0754 | 0 | 0.39 |
| 381 | 1105 | 0 | 0.39 |
| 382 | 0541 | 0 | 0.38 |
| 383 | 0908 | 0 | 0.38 |
| 384 | 0363 | 0 | 0.37 |
| 385 | 0803 | 0 | 0.37 |
| 386 | 0340 | 0 | 0.35 |
| 387 | 0476 | 0 | 0.34 |
| 388 | 0429 | 0 | 0.34 |
| 389 | 1107 | 0 | 0.33 |
| 390 | 0595 | 0 | 0.33 |
| 391 | 1064 | 0 | 0.32 |
| 392 | 0448 | 0 | 0.31 |
| 393 | 0935 | 0 | 0.31 |
| 394 | 0015 | 0 | 0.31 |
| 395 | 0828 | 0 | 0.31 |
| 396 | 0028 | 0 | 0.30 |
| 397 | 0642 | 0 | 0.30 |
| 398 | 0821 | 0 | 0.29 |
| 399 | 0324 | 0 | 0.29 |
| 400 | 0162 | 0 | 0.29 |
| 401 | 0512 | 0 | 0.29 |
| 402 | 0005 | 0 | 0.29 |
| 403 | 0477 | 0 | 0.29 |
| 404 | 0214 | 0 | 0.28 |
| 405 | 1009 | 0 | 0.28 |
| 406 | 0097 | 0 | 0.27 |
| No. | Ech_ tag no. | Mean ELISA OD_650_ | Antigenicity score |
| 407 | 0739 | 0 | 0.27 |
| 408 | 0319 | 0 | 0.27 |
| 409 | 0480 | 0 | 0.26 |
| 410 | 0131 | 0 | 0.26 |
| 411 | 0194 | 0 | 0.25 |
| 412 | 0163 | 0 | 0.25 |
| 413 | 0221 | 0 | 0.23 |
| 414 | 0792 | 0 | 0.23 |
| 415 | 1108 | 0 | 0.22 |
| 416 | 0874 | 0 | 0.22 |
| 417 | 0069 | 0 | 0.22 |
| 418 | 0843 | 0 | 0.21 |
| 419 | 0901 | 0 | 0.21 |
| 420 | 1072 | 0 | 0.20 |
| 421 | 0460 | 0 | 0.20 |
| 422 | 0229 | 0 | 0.19 |
| 423 | 0641 | 0 | 0.18 |
| 424 | 1010 | 0 | 0.17 |
| 425 | 0149 | 0 | 0.17 |
| No. | Ech_ tag no. | Mean ELISA OD_650_ | Antigenicity score |
| 426 | 0529 | 0 | 0.17 |
| 427 | 0356 | 0 | 0.15 |
| 428 | 1069 | 0 | 0.15 |
| 429 | 0623 | 0 | 0.14 |
| 430 | 0403 | 0 | 0.13 |
| 431 | 1062 | 0 | 0.13 |
| 432 | 0891 | 0 | 0.13 |
| 433 | 0714 | 0 | 0.12 |
| 434 | 0290 | 0 | 0.12 |
| 435 | 0008 | 0 | 0.11 |
| 436 | 0930 | 0 | 0.11 |
| 437 | 0007 | 0 | 0.10 |
| 438 | 0077 | 0 | 0.10 |
| 439 | 0917 | 0 | 0.10 |
| 440 | 0564 | 0 | 0.09 |
| 441 | 0511 | 0 | 0.07 |
| 442 | 1042 | 0 | 0.06 |
| 443 | 0380 | 0 | 0.05 |
| 444 | 1048 | 0 | 0.03 |

**TABLE S2** *E. canis* protein immunoreactivity ranked by mean ELISA OD values.

| No. | Ecaj_ tag no. | Mean ELISA OD_650_ | Antigenicity score |
| --- | --- | --- | --- |
| 1 | 0274 | 2.24 | 0.42 |
| 2 | 0647 | 1.65 | 0.57 |
| 3 | 0026 | 1.57 | 0.83 |
| 4 | 0642 | 1.26 | 0.46 |
| 5 | 0710 | 1.23 | 0.66 |
| 6 | 0943 | 1.23 | 0.46 |
| 7 | 0873 | 0.93 | 0.06 |
| 8 | 0305 | 0.92 | 0.37 |
| 9 | 0244 | 0.88 | 0.17 |
| 10 | 0817 | 0.83 | 0.44 |
| 11 | 0010 | 0.74 | 0.33 |
| 12 | 0862 | 0.67 | 0.25 |
| 13 | 0293 | 0.62 | 0.29 |
| 14 | 0614 | 0.60 | 0.60 |
| 15 | 0794 | 0.47 | 0.57 |
| 16 | 0534 | 0.45 | 0.10 |
| 17 | 03910 | 0.45 | 0.07 |
| 18 | 0820 | 0.45 | 0.30 |
| 19 | 0328 | 0.44 | 0.35 |
| 20 | 0560 | 0.43 | 0.06 |
| 21 | 0018 | 0.42 | 0.82 |
| 22 | 0809 | 0.42 | 0.44 |
| 23 | 05075 | 0.36 | 0.15 |
| 24 | 0548 | 0.34 | 0.70 |
| 25 | 0163 | 0.34 | 0.04 |
| 26 | 0515 | 0.34 | 0.33 |
| 27 | 0076 | 0.33 | 0.34 |
| 28 | 0879 | 0.33 | 0.40 |
| 29 | 03920 | 0.33 | 0.86 |
| 30 | 0038 | 0.32 | 0.46 |
| 31 | 0557 | 0.30 | 0.06 |
| 32 | 0034 | 0.29 | 0.23 |
| 33 | 0317 | 0.28 | 0.24 |
| 34 | 0062 | 0.26 | 0.87 |
| 35 | 0088 | 0.26 | 0.73 |
| 36 | 0533 | 0.26 | 0.29 |
| 37 | 0814 | 0.26 | 0.30 |
| 38 | 0704 | 0.23 | 0.14 |
| 39 | 0294 | 0.23 | 0.06 |
| No. | Ecaj_ tag no. | Mean ELISA OD_650_ | Antigenicity score |
| 40 | 0453 | 0.22 | 0.15 |
| 41 | 0807 | 0.21 | 0.35 |
| 42 | 0085 | 0.20 | 0.23 |
| 43 | 03915 | 0.20 | 0.08 |
| 44 | 0522 | 0.20 | 0.50 |
| 45 | 0520 | 0.19 | 0.38 |
| 46 | 0032 | 0.19 | 0.27 |
| 47 | 0030 | 0.18 | 0.59 |
| 48 | 0536 | 0.18 | 0.14 |
| 49 | 0559 | 0.18 | 0.12 |
| 50 | 0006 | 0.17 | 0.53 |
| 51 | 0697 | 0.17 | 0.09 |
| 52 | 0860 | 0.16 | 0.07 |
| 53 | 0079 | 0.16 | 0.54 |
| 54 | 0677 | 0.15 | 0.12 |
| 55 | 0874 | 0.15 | 0.42 |
| 56 | 0066 | 0.15 | 0.91 |
| 57 | 0840 | 0.15 | 0.11 |
| 58 | 0090 | 0.14 | 0.69 |
| 59 | 0581 | 0.14 | 0.09 |
| 60 | 0800 | 0.14 | 0.57 |
| 61 | 0778 | 0.14 | 0.45 |
| 62 | 0452 | 0.14 | 0.13 |
| 63 | 0841 | 0.13 | 0.05 |
| 64 | 0544 | 0.13 | 0.26 |
| 65 | 0785 | 0.13 | 0.53 |
| 66 | 0864 | 0.13 | 0.22 |
| 67 | 0039 | 0.12 | 0.08 |
| 68 | 0215 | 0.12 | 0.24 |
| 69 | 0629 | 0.12 | 0.35 |
| 70 | 0531 | 0.12 | 0.34 |
| 71 | 0883 | 0.12 | 0.16 |
| 72 | 0184 | 0.12 | 0.30 |
| 73 | 0495 | 0.11 | 0.62 |
| 74 | 0205 | 0.11 | 0.29 |
| 75 | 0019 | 0.11 | 0.69 |
| 76 | 0780 | 0.11 | 0.27 |
| 77 | 0540 | 0.11 | 0.54 |
| 78 | 0541 | 0.10 | 0.57 |
| No. | Ecaj_ tag no. | Mean ELISA OD_650_ | Antigenicity score |
| 79 | 0044 | 0.10 | 0.67 |
| 80 | 0202 | 0.10 | 0.31 |
| 81 | 0045 | 0.10 | 0.73 |
| 82 | 0073 | 0.10 | 0.89 |
| 83 | 0886 | 0.10 | 0.32 |
| 84 | 0586 | 0.10 | 0.13 |
| 85 | 0211 | 0.10 | 0.08 |
| 86 | 0682 | 0.10 | 0.11 |
| 87 | 0781 | 0.09 | 0.46 |
| 88 | 0561 | 0.09 | 0.28 |
| 89 | 0654 | 0.09 | 0.17 |
| 90 | 0813 | 0.09 | 0.27 |
| 91 | 0521 | 0.09 | 0.54 |
| 92 | 0036 | 0.09 | 0.81 |
| 93 | 0189 | 0.09 | 0.09 |
| 94 | 0224 | 0.09 | 0.26 |
| 95 | 0875 | 0.09 | 0.40 |
| 96 | 0846 | 0.09 | 0.58 |
| 97 | 0652 | 0.08 | 0.53 |
| 98 | 0631 | 0.08 | 0.30 |
| 99 | 0621 | 0.08 | 0.70 |
| 100 | 0806 | 0.08 | 0.49 |
| 101 | 0449 | 0.08 | 0.06 |
| 102 | 0640 | 0.08 | 0.47 |
| 103 | 0867 | 0.08 | 0.24 |
| 104 | 0549 | 0.08 | 0.42 |
| 105 | 0070 | 0.08 | 0.50 |
| 106 | 0089 | 0.08 | 0.39 |
| 107 | 0556 | 0.08 | 0.11 |
| 108 | 0516 | 0.07 | 0.31 |
| 109 | 0082 | 0.07 | 0.27 |
| 110 | 0093 | 0.07 | 0.53 |
| 111 | 0552 | 0.07 | 0.06 |
| 112 | 0518 | 0.07 | 0.36 |
| 113 | 0585 | 0.07 | 0.04 |
| 114 | 0712 | 0.07 | 0.50 |
| 115 | 0669 | 0.07 | 0.24 |
| 116 | 0784 | 0.07 | 0.31 |
| 117 | 0685 | 0.07 | 0.03 |
| 118 | 0690 | 0.07 | 0.13 |
| 119 | 0713 | 0.06 | 0.18 |
| No. | Ecaj_ tag no. | Mean ELISA OD_650_ | Antigenicity score |
| 120 | 0075 | 0.06 | 0.43 |
| 121 | 0852 | 0.06 | 0.16 |
| 122 | 0546 | 0.06 | 0.44 |
| 123 | 0696 | 0.06 | 0.15 |
| 124 | 0692 | 0.06 | 0.28 |
| 125 | 0855 | 0.06 | 0.22 |
| 126 | 0759 | 0.06 | 0.14 |
| 127 | 0008 | 0.06 | 0.45 |
| 128 | 0041 | 0.06 | 0.09 |
| 129 | 0046 | 0.06 | 0.42 |
| 130 | 0760 | 0.06 | 0.38 |
| 131 | 0519 | 0.06 | 0.07 |
| 132 | 0570 | 0.06 | 0.13 |
| 133 | 0042 | 0.06 | 0.19 |
| 134 | 0094 | 0.06 | 0.78 |
| 135 | 0701 | 0.06 | 0.55 |
| 136 | 0068 | 0.06 | 0.90 |
| 137 | 0027 | 0.06 | 0.39 |
| 138 | 0661 | 0.05 | 0.31 |
| 139 | 0708 | 0.05 | 0.39 |
| 140 | 0670 | 0.05 | 0.15 |
| 141 | 0885 | 0.05 | 0.21 |
| 142 | 0313 | 0.05 | 0.20 |
| 143 | 0567 | 0.05 | 0.39 |
| 144 | 0532 | 0.05 | 0.11 |
| 145 | 0801 | 0.05 | 0.37 |
| 146 | 0871 | 0.05 | 0.23 |
| 147 | 0558 | 0.05 | 0.13 |
| 148 | 0275 | 0.05 | 0.28 |
| 149 | 0573 | 0.05 | 0.36 |
| 150 | 0825 | 0.05 | 0.48 |
| 151 | 0926 | 0.05 | 0.82 |
| 152 | 0681 | 0.05 | 0.29 |
| 153 | 0892 | 0.05 | 0.50 |
| 154 | 0686 | 0.05 | 0.40 |
| 155 | 0037 | 0.05 | 0.14 |
| 156 | 0665 | 0.05 | 0.32 |
| 157 | 0087 | 0.05 | 0.16 |
| 158 | 0256 | 0.05 | 0.84 |
| 159 | 0526 | 0.05 | 0.46 |
| 160 | 0574 | 0.04 | 0.21 |
| No. | Ecaj_ tag no. | Mean ELISA OD_650_ | Antigenicity score |
| 161 | 0680 | 0.04 | 0.52 |
| 162 | 0842 | 0.04 | 0.06 |
| 163 | 0668 | 0.04 | 0.15 |
| 164 | 0709 | 0.04 | 0.17 |
| 165 | 0774 | 0.04 | 0.54 |
| 166 | 0868 | 0.04 | 0.11 |
| 167 | 0891 | 0.04 | 0.35 |
| 168 | 0876 | 0.04 | 0.37 |
| 169 | 0630 | 0.04 | 0.33 |
| 170 | 0061 | 0.04 | 0.56 |
| 171 | 0691 | 0.04 | 0.45 |
| 172 | 0086 | 0.04 | 0.27 |
| 173 | 0645 | 0.04 | 0.22 |
| 174 | 0031 | 0.04 | 0.34 |
| 175 | 0074 | 0.04 | 0.25 |
| 176 | 0635 | 0.04 | 0.61 |
| 177 | 0928 | 0.04 | 0.62 |
| 178 | 0803 | 0.04 | 0.21 |
| 179 | 0666 | 0.04 | 0.54 |
| 180 | 0684 | 0.04 | 0.25 |
| 181 | 0792 | 0.04 | 0.05 |
| 182 | 0810 | 0.04 | 0.17 |
| 183 | 0663 | 0.04 | 0.82 |
| 184 | 0688 | 0.04 | 0.53 |
| 185 | 0626 | 0.04 | 0.38 |
| 186 | 0888 | 0.03 | 0.23 |
| 187 | 0003 | 0.03 | 0.67 |
| 188 | 0575 | 0.03 | 0.17 |
| 189 | 0029 | 0.03 | 0.35 |
| 190 | 0083 | 0.03 | 0.21 |
| 191 | 0779 | 0.03 | 0.06 |
| 192 | 0207 | 0.03 | 0.53 |
| 193 | 0853 | 0.03 | 0.24 |
| 194 | 0099 | 0.03 | 0.94 |
| 195 | 0624 | 0.03 | 0.18 |
| 196 | 0659 | 0.03 | 0.44 |
| 197 | 0940 | 0.03 | 0.55 |
| 198 | 0672 | 0.03 | 0.45 |
| 199 | 0091 | 0.03 | 0.82 |
| 200 | 0706 | 0.03 | 0.70 |
| 201 | 0005 | 0.03 | 0.88 |
| No. | Ecaj_ tag no. | Mean ELISA OD_650_ | Antigenicity score |
| 202 | 0376 | 0.03 | 0.49 |
| 203 | 0776 | 0.03 | 0.32 |
| 204 | 0092 | 0.02 | 0.47 |
| 205 | 0877 | 0.02 | 0.05 |
| 206 | 0043 | 0.02 | 0.51 |
| 207 | 0243 | 0.02 | 0.47 |
| 208 | 0362 | 0.02 | 0.39 |
| 209 | 0021 | 0.02 | 0.41 |
| 210 | 0055 | 0.02 | 0.35 |
| 211 | 0095 | 0.02 | 0.07 |
| 212 | 0927 | 0.02 | 0.21 |
| 213 | 0872 | 0.02 | 0.07 |
| 214 | 0935 | 0.02 | 0.12 |
| 215 | 0096 | 0.02 | 0.86 |
| 216 | 0098 | 0.02 | 0.26 |
| 217 | 0250 | 0.02 | 0.18 |
| 218 | 0490 | 0.02 | 0.54 |
| 219 | 0893 | 0.02 | 0.41 |
| 220 | 0053 | 0.02 | 0.51 |
| 221 | 0024 | 0.02 | 0.20 |
| 222 | 0396 | 0.02 | 0.36 |
| 223 | 0757 | 0.02 | 0.04 |
| 224 | 0339 | 0.02 | 0.10 |
| 225 | 0015 | 0.02 | 0.45 |
| 226 | 0934 | 0.02 | 0.61 |
| 227 | 0221 | 0.02 | 0.46 |
| 228 | 0565 | 0.02 | 0.14 |
| 229 | 0795 | 0.02 | 0.44 |
| 230 | 0196 | 0.02 | 0.10 |
| 231 | 0380 | 0.02 | 0.49 |
| 232 | 0071 | 0.01 | 0.93 |
| 233 | 0100 | 0.01 | 0.89 |
| 234 | 0793 | 0.01 | 0.06 |
| 235 | 0025 | 0.01 | 0.11 |
| 236 | 0052 | 0.01 | 0.25 |
| 237 | 0699 | 0.01 | 0.72 |
| 238 | 0592 | 0.01 | 0.20 |
| 239 | 0327 | 0.01 | 0.50 |
| 240 | 0465 | 0.01 | 0.10 |
| 241 | 0265 | 0.01 | 0.06 |
| 242 | 0208 | 0.01 | 0.53 |
| No. | Ecaj_ tag no. | Mean ELISA OD_650_ | Antigenicity score |
| 243 | 0049 | 0.01 | 0.24 |
| 244 | 0238 | 0.01 | 0.31 |
| 245 | 0057 | 0.01 | 0.17 |
| 246 | 0022 | 0.01 | 0.83 |
| 247 | 0064 | 0.01 | 0.48 |
| 248 | 0001 | 0.01 | 0.29 |
| 249 | 0040 | 0.01 | 0.42 |
| 250 | 0223 | 0.01 | 0.17 |
| 251 | 0203 | 0.01 | 0.40 |
| 252 | 0514 | 0.01 | 0.14 |
| 253 | 0587 | 0.01 | 0.37 |
| 254 | 0929 | 0.01 | 0.44 |
| 255 | 0193 | 0.01 | 0.39 |
| 256 | 0276 | 0.01 | 0.56 |
| 257 | 0204 | 0.01 | 0.20 |
| 258 | 0235 | 0.01 | 0.39 |
| 259 | 0359 | 0.01 | 0.42 |
| 260 | 0765 | 0.01 | 0.69 |
| 261 | 0455 | 0 | 0.29 |
| 262 | 0080 | 0 | 0.26 |
| 263 | 0464 | 0 | 0.52 |
| 264 | 0097 | 0 | 0.35 |
| 265 | 0218 | 0 | 0.38 |
| 266 | 0072 | 0 | 0.90 |
| 267 | 0077 | 0 | 0.58 |
| 268 | 0473 | 0 | 0.26 |
| 269 | 0844 | 0 | 0.50 |
| 270 | 0944 | 0 | 0.62 |
| 271 | 0002 | 0 | 0.04 |
| 272 | 0004 | 0 | 0.64 |
| 273 | 0007 | 0 | 0.10 |
| 274 | 0011 | 0 | 0.24 |
| 275 | 0012 | 0 | 0.73 |
| 276 | 0013 | 0 | 0.62 |
| 277 | 0014 | 0 | 0.19 |
| 278 | 0017 | 0 | 0.72 |
| 279 | 0020 | 0 | 0.94 |
| 280 | 0023 | 0 | 0.42 |
| 281 | 0033 | 0 | 0.65 |
| 282 | 0035 | 0 | 0.17 |
| 283 | 0048 | 0 | 0.55 |
| No. | Ecaj_ tag no. | Mean ELISA OD_650_ | Antigenicity score |
| 284 | 0050 | 0 | 0.05 |
| 285 | 0051 | 0 | 0.22 |
| 286 | 0054 | 0 | 0.64 |
| 287 | 0056 | 0 | 0.39 |
| 288 | 0058 | 0 | 0.09 |
| 289 | 0059 | 0 | 0.42 |
| 290 | 0060 | 0 | 0.84 |
| 291 | 0063 | 0 | 0.94 |
| 292 | 0065 | 0 | 0.87 |
| 293 | 0067 | 0 | 0.92 |
| 294 | 0069 | 0 | 0.94 |
| 295 | 0078 | 0 | 0.20 |
| 296 | 0081 | 0 | 0.09 |
| 297 | 0084 | 0 | 0.55 |
| 298 | 0131 | 0 | 0.26 |
| 299 | 0190 | 0 | 0.10 |
| 300 | 0197 | 0 | 0.13 |
| 301 | 0200 | 0 | 0.23 |
| 302 | 0227 | 0 | 0.55 |
| 303 | 0241 | 0 | 0.27 |
| 304 | 0245 | 0 | 0.32 |
| 305 | 0251 | 0 | 0.03 |
| 306 | 0253 | 0 | 0.74 |
| 307 | 0257 | 0 | 0.41 |
| 308 | 0262 | 0 | 0.31 |
| 309 | 0263 | 0 | 0.11 |
| 310 | 0264 | 0 | 0.05 |
| 311 | 0267 | 0 | 0.28 |
| 312 | 0269 | 0 | 0.37 |
| 313 | 0270 | 0 | 0.57 |
| 314 | 0272 | 0 | 0.33 |
| 315 | 0281 | 0 | 0.53 |
| 316 | 0283 | 0 | 0.33 |
| 317 | 0284 | 0 | 0.14 |
| 318 | 0286 | 0 | 0.26 |
| 319 | 0292 | 0 | 0.47 |
| 320 | 0295 | 0 | 0.05 |
| 321 | 0296 | 0 | 0.41 |
| 322 | 0297 | 0 | 0.81 |
| 323 | 0301 | 0 | 0.91 |
| 324 | 0302 | 0 | 0.26 |
| No. | Ecaj_ tag no. | Mean ELISA OD_650_ | Antigenicity score |
| 325 | 0303 | 0 | 0.23 |
| 326 | 0304 | 0 | 0.53 |
| 327 | 0307 | 0 | 0.10 |
| 328 | 0319 | 0 | 0.55 |
| 329 | 0320 | 0 | 0.46 |
| 330 | 0322 | 0 | 0.29 |
| 331 | 0326 | 0 | 0.38 |
| 332 | 0329 | 0 | 0.26 |
| 333 | 0352 | 0 | 0.54 |
| 334 | 0353 | 0 | 0.18 |
| 335 | 0354 | 0 | 0.32 |
| 336 | 0360 | 0 | 0.25 |
| 337 | 0364 | 0 | 0.37 |
| 338 | 0367 | 0 | 0.48 |
| 339 | 0370 | 0 | 0.57 |
| 340 | 0371 | 0 | 0.36 |
| 341 | 0375 | 0 | 0.42 |
| 342 | 0381 | 0 | 0.79 |
| 343 | 0389 | 0 | 0.17 |
| 344 | 0392 | 0 | 0.34 |
| 345 | 0393 | 0 | 0.31 |
| 346 | 0397 | 0 | 0.41 |
| 347 | 0398 | 0 | 0.24 |
| 348 | 0402 | 0 | 0.52 |
| 349 | 0403 | 0 | 0.43 |
| 350 | 0405 | 0 | 0.57 |
| 351 | 0407 | 0 | 0.48 |
| 352 | 0410 | 0 | 0.32 |
| 353 | 0413 | 0 | 0.31 |
| 354 | 0418 | 0 | 0.45 |
| 355 | 0432 | 0 | 0.57 |
| 356 | 0437 | 0 | 0.40 |
| 357 | 0445 | 0 | 0.81 |
| 358 | 0458 | 0 | 0.19 |
| 359 | 0459 | 0 | 0.42 |
| 360 | 0461 | 0 | 0.23 |
| 361 | 0468 | 0 | 0.34 |
| 362 | 0469 | 0 | 0.35 |
| 363 | 0470 | 0 | 0.05 |
| 364 | 0492 | 0 | 0.27 |
| 365 | 0493 | 0 | 0.78 |
| No. | Ecaj_ tag no. | Mean ELISA OD_650_ | Antigenicity score |
| 366 | 0498 | 0 | 0.26 |
| 367 | 0501 | 0 | 0.28 |
| 368 | 0510 | 0 | 0.50 |
| 369 | 0511 | 0 | 0.08 |
| 370 | 0550 | 0 | 0.30 |
| 371 | 0562 | 0 | 0.51 |
| 372 | 0582 | 0 | 0.10 |
| 373 | 0593 | 0 | 0.05 |
| 374 | 0616 | 0 | 0.28 |
| 375 | 0618 | 0 | 0.35 |
| 376 | 0619 | 0 | 0.15 |
| 377 | 0658 | 0 | 0.77 |
| 378 | 0674 | 0 | 0.38 |
| 379 | 0790 | 0 | 0.21 |
| 380 | 0797 | 0 | 0.19 |
| 381 | 0798 | 0 | 0.47 |
| 382 | 0811 | 0 | 0.07 |
| 383 | 0854 | 0 | 0.38 |
| 384 | 0861 | 0 | 0.25 |
| 385 | 0869 | 0 | 0.26 |
| 386 | 0880 | 0 | 0.05 |
| 387 | 0884 | 0 | 0.56 |
| 388 | 0921 | 0 | 0.55 |
| 389 | 0933 | 0 | 0.37 |
| 390 | 0937 | 0 | 0.04 |
| 391 | 0939 | 0 | 0.78 |
| 392 | 05045 | 0 | 0.54 |
| 393 | 0812 | 0 | 0.26 |
| 394 | 0028 | 0 | 0.20 |
| 395 | 0579 | 0 | 0.58 |
| 396 | 0504 | 0 | 0.08 |
| 397 | 0505 | 0 | 0.31 |
| 398 | 0491 | 0 | 0.46 |
| 399 | 0553 | 0 | 0.46 |
| 400 | 0214 | 0 | 0.57 |
| 401 | 0249 | 0 | 0.24 |
| 402 | 0009 | 0 | 0.13 |
| 403 | 0340 | 0 | 0.31 |
| 404 | 0047 | 0 | 0.80 |
| 405 | 0277 | 0 | 0.13 |
|  |  |  |  |
